# Short telomere length is associated with impaired cognitive performance in European ancestry cohorts

**DOI:** 10.1038/tp.2017.73

**Published:** 2017-04-18

**Authors:** S Hägg, Y Zhan, R Karlsson, L Gerritsen, A Ploner, S J van der Lee, L Broer, J Deelen, R E Marioni, A Wong, A Lundquist, G Zhu, N K Hansell, E Sillanpää, I O Fedko, N A Amin, M Beekman, A J M de Craen, S Degerman, S E Harris, K-J Kan, C M Martin-Ruiz, G W Montgomery, NeuroCHARGE Cognitive Working Group, A N Adolfsson, C A Reynolds, N J Samani, H E D Suchiman, A Viljanen, T von Zglinicki, M J Wright, J-J Hottenga, D I Boomsma, T Rantanen, J A Kaprio, D R Nyholt, N G Martin, L Nyberg, R Adolfsson, D Kuh, J M Starr, I J Deary, P E Slagboom, C M van Duijn, V Codd, N L Pedersen

**Affiliations:** 1 Department of Medical Epidemiology and Biostatistics, Karolinska Institutet, Stockholm, Sweden; 2 Department of Epidemiology, Erasmus University Medical Center, Rotterdam, The Netherlands; 3 Department of Internal Medicine, Erasmus University Medical Center, Rotterdam, The Netherlands; 4 Department of Molecular Epidemiology, Leiden University Medical Center, Leiden, The Netherlands; 5 Max Planck Institute for Biology of Ageing, Cologne, Germany; 6 Centre for Genomic and Experimental Medicine, Institute of Genetics and Molecular Medicine, University of Edinburgh, Edinburgh, UK; 7 Centre for Cognitive Ageing and Cognitive Epidemiology, University of Edinburgh, Edinburgh, UK; 8 Queensland Brain Institute, University of Queensland, Brisbane, QLD, Australia; 9 MRC Unit for Lifelong Health and Ageing at UCL, London, UK; 10 Department of Statistics, Umeå University, Umeå, Sweden; 11 QIMR Berghofer Medical Research Institute, Brisbane, QLD, Australia; 12 Gerontology Research Center, Faculty of Sport and Health Sciences, University of Jyväskylä, Jyväskylä, Finland; 13 Department Biological Psychology, Vrije Universiteit, Amsterdam, The Netherlands; 14 Department of Gerontology and Geriatrics, Leiden University Medical Center, Leiden, The Netherlands; 15 Department of Medical Biosciences, Umeå University, Umeå, Sweden; 16 NIHR Newcastle Biomedical Research Centre & Unit, Institute of Neurosciences, Newcastle University, Campus for Ageing and Vitality, Newcastle upon Tyne, UK; 17 Department of Clinical Sciences, Umeå University, Umeå, Sweden; 18 Department of Psychology, University California Riverside, Riverside, CA, USA; 19 Department of Cardiovascular Sciences, University of Leicester, Leicester, UK; 20 National Institute for Health Research Leicester Cardiovascular Biomedical Research Unit, Glenfield Hospital, Leicester, UK; 21 Newcastle University Institute for Ageing, Institute for Cell & Molecular Biosciences, Newcastle upon Tyne, UK; 22 Centre for Advanced Imaging, University of Queensland, Brisbane, QLD, Australia; 23 Department of Public Health, Clincum, University of Helsinki, Helsinki, Finland; 24 Institute for Molecular Medicine Finland (FIMM), University of Helsinki, Helsinki, Finland; 25 National Institute for Health and Welfare (THL), Helsinki, Finland; 26 Institute of Health and Biomedical Innovation, Queensland University of Technology, Brisbane, QLD, Australia; 27 Department of Radiation Sciences, Umeå University, Umeå, Sweden; 28 Department of Integrative Medical Biology (IMB), Umeå University, Umeå, Sweden; 29 Umeå center for Functional Brain Imaging, Umeå University, Umeå, Sweden; 30 Alzheimer Scotland Dementia Research Centre, University of Edinburgh, Edinburgh, UK; 31 Department of Psychology, University of Edinburgh, Edinburgh, UK

## Abstract

The association between telomere length (TL) dynamics on cognitive performance over the life-course is not well understood. This study meta-analyses observational and causal associations between TL and six cognitive traits, with stratifications on *APOE* genotype, in a Mendelian Randomization (MR) framework. Twelve European cohorts (*N*=17 052; mean age=59.2±8.8 years) provided results for associations between qPCR-measured TL (T/S-ratio scale) and general cognitive function, mini-mental state exam (MMSE), processing speed by digit symbol substitution test (DSST), visuospatial functioning, memory and executive functioning (STROOP). In addition, a genetic risk score (GRS) for TL including seven known genetic variants for TL was calculated, and used in associations with cognitive traits as outcomes in all cohorts. Observational analyses showed that longer telomeres were associated with better scores on DSST (*β*=0.051 per s.d.-increase of TL; 95% confidence interval (CI): 0.024, 0.077; *P*=0.0002), and MMSE (*β*=0.025; 95% CI: 0.002, 0.047; *P*=0.03), and faster STROOP (*β*=−0.053; 95% CI: −0.087, −0.018; *P*=0.003). Effects for DSST were stronger in *APOE* ɛ4 non-carriers (*β*=0.081; 95% CI: 0.045, 0.117; *P*=1.0 × 10^−5^), whereas carriers performed better in STROOP (*β*=−0.074; 95% CI: −0.140, −0.009; *P*=0.03). Causal associations were found for STROOP only (*β*=−0.598 per s.d.-increase of TL; 95% CI: −1.125, −0.072; *P*=0.026), with a larger effect in ɛ4-carriers (*β*=−0.699; 95% CI: −1.330, −0.069; *P*=0.03). Two-sample replication analyses using CHARGE summary statistics showed causal effects between TL and general cognitive function and DSST, but not with STROOP. In conclusion, we suggest causal effects from longer TL on better cognitive performance, where *APOE* ɛ4-carriers might be at differential risk.

## Introduction

Telomeres, short DNA sequences at the end of chromosomes, are considered markers of biological age. Cell replication and oxidative stressors contribute to the loss of telomere nucleotides over time; below critical length, cellular senescence will follow.^1^ An increasing number of studies have shown the importance of telomere length (TL) in ageing, specifically in the development of dementia and cognitive impairment.^2, 3, 4, 5, 6, 7, 8^ Using a relatively large group of non-demented older individuals, Yaffe *et al.*^3^ demonstrated an association between longer telomeres and higher score in the digit symbol substitution test (DSST)—a measure of processing speed—at baseline. After seven years, the individuals with longer telomeres at baseline performed better in the modified mini-mental state exam (MMSE) but not in DSST. Cohen-Manheim *et al.*^8^ investigated TL in young adults and found faster attrition rates with poorer mid-life general- and domain-specific cognitive performance but no association with baseline TL. In addition, a cross-sectional study of non-demented individuals concluded that *APOE* ɛ4-carriers had longer TL but faster attrition rates indicating abnormal cell turnover.^9^ Other studies have also shown associations between TL and cognition with conflicting results or were underpowered.^4, 6, 10, 11^ Hence, the underlying mechanisms by which telomeres may be involved in cognitive performance are complex, and larger efforts are needed to elucidate this relationship. Moreover, it is still unclear whether short telomeres are a cause, consequence or both for cognitive impairment.

One way to predict a causal association is to conduct a Mendelian Randomization (MR) study,^12^ in which genetic markers are used as proxies for an exposure (TL), to investigate an un-biased effect on an outcome (cognitive performance). Because genetic variants are randomly assorted at meiosis, they are generally free from conventional confounding and hence the MR study design is often referred to as nature's own clinical trial.^13^

We hypothesized that TL is an indicator of cellular stability, which as such affects functioning throughout the body, including performance on all types of cognitive traits.^14^ In addition, individuals carrying the *APOE* ɛ4 allele are more susceptible to cognitive impairment and are therefore of special interest.^15^ The objective of our study was to conduct a meta-analytic MR study of the association between TL and six cognitive traits in 12 European ancestry cohorts (*N*=17 052). A secondary aim was to stratify on *APOE* ɛ4 genotype to investigate if carriers were at different risks given their worse cognitive ability. Most cohorts were enrolled through the European Network of Genomic and Genetic Epidemiology (ENGAGE) Consortium. Telomere measurements were performed by qPCR and a genetic risk score (GRS) with seven genetic variants associated with TL^16^ was calculated. Observational- as well as causal estimates were subsequently obtained using an MR design.^17^ In a replication effort, summary statistics from the Cohorts for Heart and Aging Research in Genomic Epidemiology (CHARGE) Consortium for the genetic associations with three cognitive traits^18, 19^ were included in a two-sample MR approach.^20^

## Materials and methods

### Study samples

Twelve cohorts with a total of 17 052 individuals (Table 1), all with European ancestry populations, participated in the ENGAGE effort. The sample-size weighted mean of age was 59.2 years with s.d.=8.8. Most cohorts contributed data measured at mid-life or older. The Leiden Longevity Study 2 (LLS2) was the oldest cohort (mean age=93.3 years). The Netherlands Twin Register (NTR) included middle-aged adults (mean age=40.3 years, s.d.=16.4) and QIMR (Twin studies at the Queensland Institute of Medical Research) included adolescents only (mean age=14.1 years, s.d.=2.4). All but one study showed a fairly even proportion of sexes (range: 49–67% women); FITSA (The Finnish Twin Study on Ageing) included women only. Additional study-specific details are found in Supplementary Table 1.

### Cognitive traits

Six different cognitive traits were tested in a combined meta-analysis of the ENGAGE cohorts: (1) general cognitive function; (2) MMSE; (3) processing speed with DSST or the variant symbol digit substitution task; (4) visuospatial functioning with block design test (BLOCK); (5) episodic memory by either verbal learning or picture learning tests (MEMORY); and (6) executive functioning using Stroop interference score (STROOP). Detailed descriptions of the different cognitive traits are found in the supplement (Supplementary Table 3). All cohorts participated with at least one cognitive trait; no single cohort had all of them (Table 1).

### Telomere length measurements

Telomere length was measured in leukocytes in whole blood/buffy coat except for the HRS study, which used measurements from saliva. DNA from saliva derives for the most part (~74%) from leukocytes,^21^ and TL measurements from blood and saliva have been reported to have good correlations (*R*=0.72).^22^ Standard qPCR techniques for TL measurement were applied as described by Cawthon^23^ with minor modifications in the Lothian Birth Cohort (LBC)^11^ and BETULA.^24^ In brief, telomere (T) and single copy gene (S) quantity were measured and a T/S-ratio was calculated. One or several reference samples were included in all runs and a relative telomere length was calculated for each sample.

### Genotyping

Information on genotyping platform, quality control and single-nucleotide polymorphisms (SNPs) used in each cohort is available in the supplement (Supplementary Data and Supplementary Table 2). An additive un-weighted GRS was calculated for each individual by summarizing the number of risk alleles from seven different loci (*TERC, TERT, NAF1, OBFC1, ZNF208, RTEL1* and *ACYP2*) where SNPs (rs10936599, rs2736100, rs7675998, rs9420907, rs8105767, rs755017 and rs11125529) have been found to associate with TL.^16^ Investigations of possible pleiotropic effects from the different genetic variants used in the GRS are discussed in the supplement (Supplementary Data). Four cohorts (ERF (Erasmus Rucphen Family), LLS, NTR and QIMR) from the current effort contributed to the original genome-wide association study (GWAS) of TL (see Supplementary Data for further discussions on implications to our study). No associations between any of the seven TL genes and cognitive traits have been tested thus far as judged from the GWAS Catalog.^25^ Genetic variants were prioritized as (1) directly genotyped, (2) imputed with good quality, (3) proxies with *r*^2^>0.8 and (4) imputed from summary statistics. The weighted mean of the GRS was 8.55 with s.d.=1.51 (Table 1). *APOE* genotype was assessed separately and available in most cohorts (Table 1).

### Replication data

Summarized results from the CHARGE Consortium's meta-analyses of genome-wide association studies between genotypes and general cognitive function (*N*=53 949),^18^ processing speed by a meta-analysis of four tests of processing speed including the DSST (*N*=32 088)^19^ and executive functioning (STROOP; *N*=7726)^19^ were used to assess causal associations from the same seven TL associated genetic variants.^16^ The CHARGE cohorts were all of European ancestry and participants were aged 45 years or older. Some ENGAGE cohorts contributed to CHARGE analyses of general cognitive function: BETULA1, ERF, HRS, and LBC1936; DSST and STROOP: ERF; and DSST: LBC1936 (Table 1; see Supplementary Data for further discussions on implications to our study). The general cognitive function phenotype was created as a composite score of multiple cognitive tests^18^ from principal component analysis. The processing speed variable was created from DSST and three similar tests, and executive functioning was assessed by either Trail Making tests or Stroop color and word interference tests.^19^ Effect sizes were missing for DSST and STROOP; hence results were presented in *Z*-scores only.

### Statistical analyses

**Figure 1:**
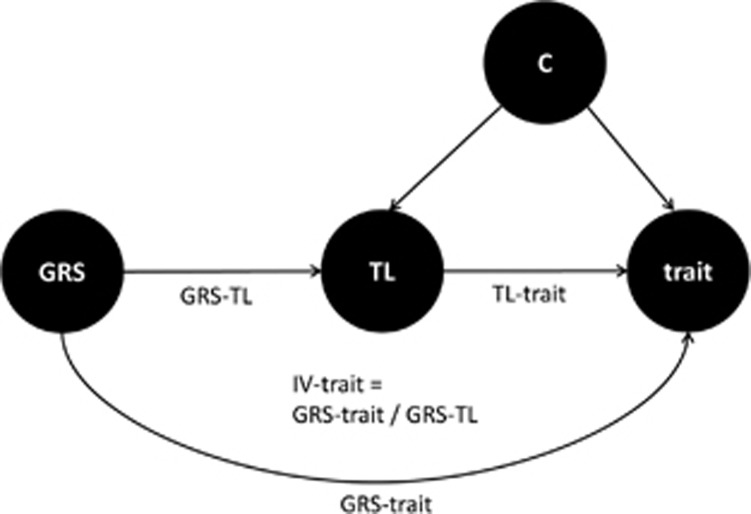
Graph describing the design of the study. A genetic risk score (GRS) for telomere length (TL) is used in the instrumental variable (IV) analysis to determine the predicted effect of TL on different cognitive traits (IV-trait). The Mendelian Randomization design allows for calculation of an estimate independent of confounders (C) in comparison to the observed effect estimated between TL and cognitive traits (TL-trait).

All variables (TL and cognitive traits) were *Z*-transformed with subtraction of the mean and division of s.d. to enable comparisons across cohorts. Age groups, instead of continuous age, were defined and used as covariates to allow for non-linear age effects: (1) 0–29 years; (2) 30–59 years; (3) 60–79 years; and (4) 80+ years. Cohorts with information on *APOE* genotype performed additional analyses stratified on ɛ4-carriers (ɛ4/ɛ4 and ɛ4/ɛ3) and non-carriers (ɛ3/ɛ3, ɛ2/ɛ3 and ɛ2/ɛ2). Individuals with the genotype ɛ2/ɛ4 are excluded from the analysis. All models are described in detail in the Supplementary Data; briefly, all cohorts contributed with summary data from three different models as depicted in Figure 1. Linear regressions were fitted for the associations of (1) TL on cognitive trait (TL-trait), (2) GRS on TL (GRS-TL), and (3) GRS on cognitive trait (GRS-trait). All models were adjusted for sex, age group and study-specific covariates. Effect estimates from all models were pooled across cohorts via fixed-effect meta-analysis, except when evidence was found for statistically significant heterogeneity (*P*-value<0.05), in which case a random-effects meta-analysis was performed instead. Then, instrumental variable (IV) analysis was conducted by calculating a Wald-type causal estimate for the effect of TL on each cognitive trait (IV−trait=GRS−trait/GRS-TL). Effect differences between observed (TL-trait) and causal/predicted (IV-trait) estimates were calculated by subtracting the causal beta from the observational beta in a Z-test (Supplementary Data). Stratified analyses on *APOE* genotype were done similarly. For summary statistics data from CHARGE, a causal estimate was calculated as described by Burgess *et al.*^20^ (Supplementary Data). Crude *P*-values are presented for all associations, that is, no multiple testing corrections have been applied.

## Results

### Observational analyses of telomere length and cognitive traits

**Figure 2:**
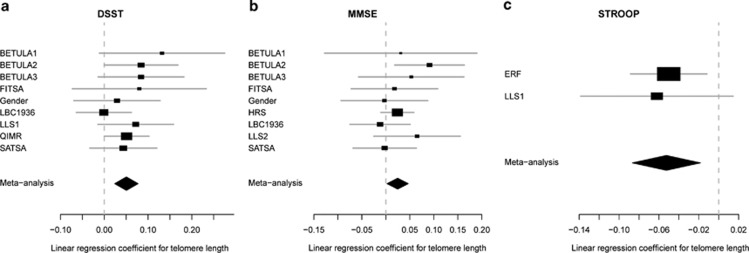
Observed effects between telomere length and cognitive traits. Fixed-effects meta-analyses were performed across cohorts and domains for all cognitive traits. Significant effects (s.d.-change in cognitive score for an s.d.-change in telomere length (TL)) were found for (**a**). digit symbol substitution test tapping processing speed (DSST), (**b**). Mini-mental state exam (MMSE), and (**c**). Stroop interference score tapping executive functioning (STROOP). All models were adjusted for age group, sex and study-specific covariates.

Significant associations, supporting the relationship between longer telomeres and better cognitive ability, were seen between TL and MMSE, DSST and STROOP using fixed-effects meta-analysis (Table 2, Figure 2). Positive associations were observed for MMSE (0.025 per s.d.-increase in TL; 95% confidence interval (CI) 0.002, 0.047) and DSST (0.051; 95% CI 0.024, 0.077). For STROOP, a negative beta (−0.053 per s.d.-increase in TL; 95% CI −0.087, −0.018) was seen, which was in accordance with the hypothesis that longer telomeres are associated with shorter time for completion of the Stroop interference test. However, after corrections for multiple comparisons, the association with MMSE was not significant.

### Genetic risk score for telomere length

The combined effect of the GRS on TL was −0.048 s.d.−change of TL per allele (95% CI: −0.064, −0.032, *P*-value=4.0*10^−9^) calculated using random-effects meta-analysis (Supplementary Data: Supplementary Figure S1). The corresponding F-statistic was 36, indicating that the GRS-TL estimate provided a sufficiently strong instrument for further use in IV analyses.^26^ Although heterogeneity was detected, all cohorts showed negative effect sizes ranging from −0.01 to −0.13 (Supplementary Table S1). Additional tests investigating possible pleiotropic effects for SNP-trait associations were done and found no evidence of such (Supplementary Data).

### Instrumental variable analyses of telomere length and cognitive traits

Instrumental variable analyses for causal associations of TL on cognitive performance were conducted for all cognitive traits. Only the association between TL and STROOP was found to be causal (Table 2), and for each s.d.-decrease in TL an effect change of −0.60 in Stroop score was detected (95% CI: −1.12, −0.07, *P*-value=0.026). However, the association would not be significant after multiple testing adjustments. The difference in effect sizes between observational and causal betas for STROOP was statistically significant (Table 2).

### Stratified analyses

Stratified meta-analyses were performed for *APOE* ɛ4-carriers (*n*⩽2380) and ɛ4 non-carriers (*n*⩽5669) separately (Supplementary Data). Observational associations were seen between TL and DSST in non-carriers (*β*=0.081, 95% CI: 0.045, 0.117, *P*-value=1 × 10^−5^) and with better performance for STROOP in carriers (*β*=−0.074, 95% CI: −0.140, −0.009, *P*-value=0.027) (Supplementary Table S4). A causal association between long telomeres and better performance for STROOP was detected amongst *APOE* ɛ4-carriers (*β*=−0.70, 95% CI: −1.33, −0.07, *P*-value=0.030), although the finding would not hold after multiple testing adjustments. No other causal effects for TL on cognitive traits were seen in either carriers or non-carriers (Supplementary Table S6).

### Replication analyses

In replication efforts, two-sample MR analyses were carried out using summary statistics from CHARGE GWAS on general cognitive function, DSST and STROOP. Data from the TL GWAS were used for the genetic instrument (Supplementary Data). Results provided evidence for a causal association between longer leukocyte TL and better general cognitive function (*β*=0.086 per s.d.-increase of TL, 95% CI: 0.016–0.156, *P*-value=0.016, Table 2) and better DSST scoring (*Z*-score=2.02, *P*-value=0.043, Table 2). No evidence for a causal effect by TL on STROOP was found (Table 2).

## Discussion

In the present study, we provide evidence for observational and causal associations between longer telomeres and better cognitive performance. By conducting a large meta-analysis of 12 cohorts from European ancestry populations with measured telomeres and assessments of cognitive function, we were able to observe associations between TL and better scoring on MMSE, DSST and STROOP. Moreover, *APOE* ɛ4*-*carriers seemed to have different effects for the observed association with worse performance in DSST but better in STROOP. The association between longer telomeres and faster completion of the Stroop interference test was also found to be significant in causal analysis for all individuals and in *APOE* ɛ4*-*carriers only. However, none of the significant causal associations detected for STROOP passed multiple testing corrections. Hence, in line with this, using summary data from CHARGE, we found support for a causal association from TL on general cognitive function and DSST, but not on STROOP.

In the biology of aging, telomere length has long been considered as a biomarker reflecting the underlying cellular state. Recently, however, several research papers have presented evidence of telomeres being involved in the process of cellular senescence causing increased risk of disease.^5, 16, 27^ Moreover, other studies suggest telomeres might even elongate in somatic cells to maintain cellular stability,^21, 28, 29^ although this phenomenon could be partly explained by leukocyte turnover or imprecise measurements. Nevertheless, telomere biology has implications for the aging processes and studies are warranted to elucidate the full complexity.

With this effort we demonstrate several observational associations between TL and cognitive traits, both confirming earlier studies and presenting new links. General cognitive function is usually operationalized as a composite score across a number of diverse cognitive domains capturing most of the cognitive variation.^18^ As an overall measure of cognition it also predicts mortality;^30^ likewise, the length of telomeres can be used to predict mortality.^31, 32^ Thus, if both general cognitive function and TL serve as valid biomarkers of aging, associations between these markers are expected, although causality needs to be further investigated. In the ENGAGE data (*N*=12 283), we were not able to detect any association between general cognitive function and TL, but using the CHARGE summary data (*N*=53 949) we provided evidence for a causal association. It is likely that the ENGAGE analysis was low in power; effect sizes had overlapping CI's. A possible biological mechanism for a causal association could be explained by overall body frailty; the lengths of telomeres are important for maintaining cellular stability at old age and hence also important for biological aging processes such as decline in cognitive performance.^1^ Causal links have also been demonstrated using animal models. A mouse with telomerase deficiency, expressing accelerated aging with malfunctioning tissue repair and impaired neurological function, had restored functions again upon telomerase reactivation.^33^

The DSST test assesses processing speed required to translate a code of symbols and digits as fast as possible in a given time frame. Processing speed has been demonstrated to have a steady, almost linear decline with advancing age, and its decline leads other forms of cognitive decline.^34, 35^ Hence, in light of this it is not surprising that we, and others,^3^ detect a fairly stable observational association of longer telomeres and better DSST scoring. The MR analysis did not indicate a causal association in our samples (*N*=4419); on the other hand, when increasing power using CHARGE data (*N*=32 088) we were able to find support for a positive causal relationship from longer TL on DSST scoring. Moreover, *APOE* ɛ4 non-carriers scored better on the test with a larger effect size seen in observational analysis from TL on DSST. Thus, as *APOE* genotype is important for elucidating different risk groups for many age-related phenotypes, it is possible that it applies to TL dynamics and cognitive performance as well.

Yaffe *et al.*^3^ showed that longer TL at baseline gave less longitudinal decline in MMSE, and we presented cross-sectional evidence from observational associations in line with these findings; longer TL is consistent with better MMSE scoring. However, while causal estimates support these associations, the CI's were wide and results did not reach statistical significance. Unfortunately, CHARGE data on MMSE were not available for replication analysis.

The STROOP variable taps the executive functioning by a combined color and word test to be completed as quickly as possible. To the best of our knowledge, there has been only one earlier small study investigating baseline and attrition TL associations with executive functioning, with inconclusive results.^8^ Our ENGAGE analysis included 2940 individuals where we found both an observational and causal association between longer telomeres and faster completion of the Stroop test. The large effect size difference was however disturbing and not explained by additional adjustments for smoking and alcohol (Supplementary Data). Further, causal associations did not hold after multiple testing corrections and when using the two-sample approach including the larger CHARGE data (*N*=7726) we could not replicate the association, although the effects were in the same direction. Hence, it is possible that the STROOP finding observed in the ENGAGE data is a false discovery. In addition, the stratified analyses by *APOE* ɛ4 genotype found ɛ4-carriers to perform better, which is contradictory to what would be expected.

The strength of this study is the effort of combining multiple European cohorts with TL, cognitive and genetic data available as well as *APOE* genotype. By doing so, we were able to detect patterns of associations for different cognitive traits that would not be possible to find in single study analyses. Moreover, we included large-scale CHARGE GWAS data sets to perform two-sample MR analyses as replication. The weaknesses of the study include generalizability, as the analyses were performed solely in European ancestry populations, and some of the cohorts were included in both ENGAGE and CHARGE analyses as described in the supplement. Moreover, heterogeneity due to different tissues used (blood and saliva) and lab-specific technical variances (TL estimates from all 12 cohorts were done in five different labs) may have driven the results toward null. Another limitation relates to the three assumptions for conducting MR studies, which have been considered as follows: (1) a strong genetic instrument should be demonstrated between the GRS and TL (GRS-TL) which we have (F-statistic=36); (2) the genetic instrument should not be confounded by e.g., age and sex (unlikely considering the randomization of alleles at conception); and (3) pleiotropic effects (when other pathways exist from the TL SNPs to the outcome (cognitive trait) without going through the intermediate phenotype (TL)) from the SNPs included in the GRS should be ruled out as much as possible. We did not find evidence for pleiotropic effects (Supplementary Data). Finally, also worth mentioning are the relatively weak *P*-values for some of the associations. The observational association for MMSE would not hold after Bonferroni correction of the *P*-value for the six cognitive traits tested, likewise for the causal associations found for STROOP.

To conclude, this study demonstrates an overall picture of the importance of biological aging processes such as TL dynamics for maintaining cognitive function throughout life. More specifically, we were able to show observational as well as causal associations between TL and different cognitive traits that have never been elucidated before. Hence, the current effort presents new important pieces of evidence for the continued search for a better understanding of the biology behind aging and the factors explaining healthy aging.

## Figures and Tables

**Table 1:** Cohorts participating in the ENGAGE study

*Cohort*	*Full name of cohort*	N	*Age (yr) mean (s.d.)*	*TL (T/S-ratio) mean (s.d.)*	*Women (%)*	*GRS mean (s.d.)*	*MMSE*	*DSST*	*BLOCK*	*MEMORY*	*STROOP*	*General cognition*	*APOE genotype*
BETULA1	The Betula Study^a^	163	50.9 (7.8)	1.01 (0.17)	58.3	8.5 (1.57)	×	×	×	×		×	Yes
BETULA2	The Betula Study^a^	396	62.4 (14.5)	0.93 (0.15)	54.8	8.87 (1.56)	×	×	×	×		×	Yes
BETULA3	The Betula Study^a^	315	60 (15)	0.97 (0.17)	54.0	8.65 (1.51)	×	×	×	×		×	Yes
ERF	Erasmus Rucphen Family (EUROSPAN)	2502	51.6 (15.8)	1.76 (0.36)	55.6	8.65 (1.57)				×	×	×	Yes
FITSA	The Finnish Twin Study on Ageing	429	68.6 (3.4)	0.9 (0.19)	100.0	8.52 (1.46)	×	×					Yes
Gender	Sex differences in health and aging	466	74.5 (2.6)	0.68 (0.15)	49.0	8.43 (1.34)		×	×	×		×	Yes
HRS	Health and Retirement Study	4117	70.4 (9.4)	1.3 (0.3)	58.0	8.6 (1.52)	×			×		×	
LBC1936	Lothian Birth Cohort	999	69.6 (0.8)	1.3 (0.5)	49.0	8.36 (1.56)	×	×	×	×		×	Yes
LLS1	Leiden Longevity Study 1	2305	59.2 (6.8)	1.46 (0.26)	54.8	8.47 (1.52)		×		×	×		Yes
LLS2	Leiden Longevity Study 2	868	93.3 (2.6)	1.28 (0.22)	61.6	8.44 (1.57)	×						Yes
NSHD	National Survey of Health and Development	2425	53 (0)	1.54 (0.91)	50.0	8.55 (1.4)				×		×	Yes
NTR	Netherlands Twin Register	200	40.3 (16.4)	2.72 (0.56)	66.5	8.5 (1.56)						×	
QIMR	Twin studies at the Queensland Institute of Medical Research	1280	14.1 (2.4)	3.7 (0.6)	52.6	8.5 (1.5)						×	
SATSA	Swedish Adoption/Twin Study of Aging	587	68.8 (9.6)	0.76 (0.27)	58.0	8.45 (1.45)		×	×	×		×	Yes

Abbreviations: BLOCK, block design test; CHARGE, Cohorts for Heart and Aging Research in Genomic Epidemiology Consortium; DSST, digit symbol substitution test; ENGAGE, European Network for Genetic and Genomic Epidemiology; GRS, genetic risk score; MEMORY, verbal memory or picture learning test; MMSE, mini-mental state exam; STROOP, stroop color word task interference score; TL, telomere length; Yr, year.

a BETULA1 and BETULA2-3 were genotyped at two different facilities.

**Table 2:** Associations between predicted and observed telomere length and different cognitive traits

*Cognitive trait*	*ENGAGE*^a^			*CHARGE*		
	*Predicted effect (IV-trait)*			*Predicted effect (IV-trait)*		
	N	*Beta (95% CI)*	P-*value*	N	*Beta (95% CI)*	P-*value*
MMSE	7066	0.291 (−0.05, 0.631)	0.095			
DSST	4419	−0.016 (−0.437, 0.405)	0.941	32 088	2.021^b^	0.043
BLOCK	5001	−0.192 (−0.594, 0.21)	0.349			
MEMORY	13 060	−0.022 (−0.264, 0.22)	0.860			
STROOP	2940	−0.598 (−1.125, −0.072)	0.026	7726	−0.780^b^	0.435
General	12 283	0.039 (−0.229, 0.306)	0.778	53 949	0.086 (0.016, 0.156)	0.016
				
*Cognitive trait*	*Observed effect (TL-trait)*					
	*Diff-*P-*value*	*Beta (95% CI)*	P-*value*			
MMSE	0.13	0.025 (0.002, 0.047)	0.030			
DSST	0.76	0.051 (0.024, 0.077)	0.0002			
BLOCK	0.34	0.004 (−0.024, 0.032)	0.781			
MEMORY	0.79	0.011 (−0.005, 0.028)	0.187			
STROOP	0.04	−0.053 (−0.087, −0.018)	0.003			
General	0.89	0.020 (−0.008, 0.047)	0.156			

Abbreviations: BLOCK, Block-design test; CI, confidence interval; Diff-*P*-value, tests for difference in estimators between observed and predicted effects; DSST, Digit-symbol substitution test; General, General cognitive performance; IV, instrumental variable; MEMORY, Verbal memory or Picture learning test; MMSE, Mini-mental state exam; STROOP, Stroop color word task interference score; TL, telomere length.

a All models are adjusted for age group and sex. No adjustments for multiple testing have been done on the reported *P*-values.

b *Z*-scores, effect sizes are missing.
