# Supplementary material for: Short telomere length is associated with impaired cognitive performance in European ancestry cohorts
Source: Transl Psychiatry. 2017 Apr 18;7(4):e1100–. doi: 10.1038/tp.2017.73 (PMC5416710; doi:10.1038/tp.2017.73)

# Short telomere length is associated with impaired cognitive performance in European ancestry cohorts

Sara Hägg et al.

Supplementary Data

[Genotype data 2](#_Toc473894789)

[Data overlap 2](#_Toc473894790)

[ENGAGE cohorts and telomere length GWAS 2](#_Toc473894791)

[CHARGE data and telomere length GWAS 2](#_Toc473894792)

[ENGAGE cohorts and CHARGE data 2](#_Toc473894793)

[Genetic risk score calculation 3](#_Toc473894794)

[Directly genotyped SNPs 3](#_Toc473894795)

[Missing and imputed genotypes 3](#_Toc473894796)

[Calculation of individual genetic risk score 3](#_Toc473894797)

[Pleiotropy test 3](#_Toc473894798)

[Functions of selected SNPs and Genes 4](#_Toc473894799)

[Model definitions 4](#_Toc473894800)

[Model I 4](#_Toc473894801)

[Model II 4](#_Toc473894802)

[Model III 4](#_Toc473894803)

[Meta-analyses 5](#_Toc473894804)

[Meta-regression analyses for age 5](#_Toc473894805)

[Instrumental variable analyses 5](#_Toc473894807)

[Stratifications 5](#_Toc473894808)

[Model I 6](#_Toc473894809)

[Model III 6](#_Toc473894810)

[Charge data analyses 6](#_Toc473894811)

[Mendelian randomization testing using Z-score 6](#_Toc473894812)

[Z-score based Wald method for a single SNP 6](#_Toc473894813)

[Testing the association between genetic risk score and the outcome based on Z-score 7](#_Toc473894814)

[Testing the association between the exposure and the outcome based on Z-score 7](#_Toc473894815)

[Acknowledgments 8](#_Toc473894816)

[References 21](#_Toc473894817)

[Figure Legends 22](#_Toc473894818)

##

## Genotype data

Genotyping was done on the Illumina Human610-Quad BeadChip (ERF, QIMR, LBC1936), the HumanOmniExpress BeadChip (BETULA1-3, LLS1, LLS2, HRS), HumanCoreExome (FITSA), HumanExome-12v1-1_A (BETULA2,3), the Metabochip (NSHD, Gender, SATSA) (Illumina Inc, San Diego, California), and on the Affymetrix 6.0 (NTR). Quality control for genotyping in each cohort was done for 1) samples: those samples with genotyping call rate <95% (or similar cohort specific cut-off) or with extreme heterozygosity were removed; and 2) SNPs: those SNPs with call rate <95% (or similar cohort specific cut-off), minor allele frequency <1% or Hardy Weinberg equilibrium p-value <10^-4^ were excluded. Imputation was performed either using a hidden Markov model algorithm implemented in MACH ([Center for Statistical Genetics, University of Michigan, Ann Arbor]) or IMPUTE 2.0 with exclusion of poorly imputed SNPs (MACH: r2hat<0.3; IMPUTE: proper_info<0.4).

## Data overlap

In the current ENGAGE effort we include data from several cohorts of European ancestry populations from around the world. Moreover, we also include data from the CHARGE consortium as well as rely on earlier telomere length GWAS data. Hence, it is inevitable that some data overlap exist between different studies and data sets. Here we try to disentangle what the implications are; essentially we have three types of overlap:

### ENGAGE cohorts and telomere length GWAS

About 40% of the ENGAGE samples in this study (ERF, LLS, NTR, QIMR) were already used to identify the seven SNPs in the TL GWAS by Codd et al[^1^](#_ENREF_1). This corresponds to the overlap between discovery sample and MR sample discussed by Lawlor[^2^](#_ENREF_2); this is not an issue for a true two-sample MR, and in our study, which is somewhere between a two-sample and a one-sample study, it would likely bias the causal estimation towards the null. This may affect the power of our study somewhat, but no impact on the validity of the results.

### CHARGE data and telomere length GWAS

In short, the same situation applies as described above which is sometimes referred to as “Winners curse”. Hence, the sample overlap was found for ERF only and would here only push our results towards the null where the validity of the results would not be questioned.

### ENGAGE cohorts and CHARGE data

Some ENGAGE studies contributed to the CHARGE cognition GWAS efforts as well. For general cognitive function BETULA1, ERF, HRS, and LBC1936 contributed with data, where LBC1936 also contributed to the DSST GWAS. The effect is moderate for both general cognitive function and DSST, where about 10-15% of the CHARGE samples have already been used in ENGAGE MR analysis. The situation is different for STROOP, where about 45% of the CHARGE data (ERF) have already been used in ENGAGE MR study.

## Genetic risk score calculation

An individual *non-weighted* genetic risk score (GRS) using the 7 SNPs reported by Codd *et al*[*^1^*](#_ENREF_1) was used as an instrumental variable (IV) in the Mendelian Randomization analysis. All cohorts with genotype information (direct or imputed for lead SNP or proxies) on *at least 4 of the 7 SNPs* listed in Table S2 performed these analyses. In short, the calculation was done using information from (in prioritized order): 1) directly genotyped lead SNPs counting 0, 1 or 2 alleles of the TL-decreasing allele; 2) imputed lead SNPs calculating the probability of the TL-decreasing allele on a scale from 0-2; 3) a directly genotyped proxy (in high linkage disequilibrium, r^2^ >0.8 in HapMap II CEU with the lead SNP) counting 0, 1 or 2 alleles of the TL-decreasing allele; or, if data was missing, 4) the average allele frequency in European populations as reported by Codd et al[^1^](#_ENREF_1). In reality, most cohorts contribute with the same SNPs, either directly genotyped or imputed. Only for two SNPs, there was a proxy used for analysis.

### Directly genotyped SNPs

Each directly genotyped SNP was transformed to be a count of 0/1/2 alleles of the TL-decreasing allele from **Table S2**.

### Missing and imputed genotypes

For SNPs that were not directly genotyped and/or had missing information (either for one individual or for the full cohort) there were three different options to complete the dataset.

Alternative 1. For imputed SNP data with good imputation quality (IMPUTE proper_info>=0.4 MACH r2hat>=0.3) the imputed lead SNP was used. The output from the imputation was different depending on which software used (posterior probabilities range 0-1 [IMPUTE] or range 0-2 [MACH]). The dosage for the TL-decreasing allele was used for calculating the score. For MACH users, the dosage information reported was used directly for calculation of the genotype score if minor allele= TL-decreasing allele. Otherwise the formula: dosage [TL-decreasing allele]=2 - dosage [non-TL-decreasing allele] was used. For IMPUTE users, the dosage was calculated as: dosage[TL-decreasing allele]= 1 * *p*(AB) + 2 * *p*(BB) where *p*(AB) and *p*(BB) were the posterior probabilities of the heterozygote (AB) and minor homozygote(BB) respectively and B was the TL-decreasing allele.

Alternative 2. A proxy with r^2^ >0.8 was used with the same effect allele as the TL-decreasing allele of the lead SNP assigned 0/1/2.

Alternative 3. The imputed SNP genotype score from the *Codd et al*[*^1^*](#_ENREF_1) paper was used.

### Calculation of individual genetic risk score

The individual score was then calculated by adding the number of TL-decreasing alleles for each individual. The sum was within the range of 0-14 (**Table S1**).

## Pleiotropy test

To assess the potential pleiotropic effects of selected SNPs, we used a method that utilized summarized statistics for this purpose. This test was conducted for each SNP set under the null hypothesis that each SNP used for the genetic risk score has an association with cognitive performance that is proportional to its association with telomere length. The rejection of the null hypothesis indicated heterogeneity of the associations between the SNPs and cognitive performance. In such cases where the null hypothesis was rejected, stepwise removal of SNPs from the SNP set was performed until there was no significant heterogeneity[^3^](#_ENREF_3)^,^ [^4^](#_ENREF_4). However, our analyses showed that there was no significant evidence supporting the pleiotropic effects in any of these SNPs (All P values > 0.1, Supplementary Figure 3). Similarly, additional sensitivity analyses using MR Egger regression and median weighted MR methods for general cognitive performance using CHARGE data did not indicate pleiotropic effects.

## Functions of selected SNPs and Genes

The rs10936599 is within ***TERC*** (Telomerase RNA component) and the rs2736100 is within ***TERT*** (Telomerase reverse transcriptase). Telomerase is a ribonucleoprotein polymerase that maintains telomere ends by addition of the telomere repeat TTAGGG. The enzyme consists of a protein component with reverse transcriptase activity, and an RNA component, encoded by this gene, that serves as a template for the telomere repeat. Telomerase expression plays a role in cellular senescence, as it is normally repressed in postnatal somatic cells resulting in progressive shortening of telomeres. Studies in mouse suggest that telomerase also participates in chromosomal repair, since de novo synthesis of telomere repeats may occur at double-stranded breaks. The gene ***RTEL1*** (Regulator of telomere elongation helicase 1), rs755017 is located within, encodes a DNA helicase which functions in the stability, protection and elongation of telomeres and interacts with proteins in the shelterin complex known to protect telomeres during DNA replication. The ***NAF1*** (Nuclear assembly factor 1 ribonucleoprotein) gene, rs7675998 is located in is involved in TERC biogenesis. The proteins coded by ***OBFC1*** (Oligonucleotide/oligosaccharide-binding fold containing 1), are subunits of an alpha accessory factor that stimulates the activity of DNA polymerase-alpha-primase, the enzyme that initiates DNA replication. OBFC1 also appears to function in a telomere associated complex with C17ORF68 and TEN1. The exact roles of ***ZNF208*** (rs8105767) and ***ACYP2*** (rs11125529) in telomere regulation are yet not clear.

## Model definitions

The following models were used to assess the relationship between (I) TL and cognitive trait, (II) genetic risk score (GRS) and TL, and (III) between GRS and cognitive trait. Study specific covariates below include study centres, sub-cohorts, family relatedness adjustments and other study specific covariates. All models were further adjusted for sex and age group defined as:

1. Age group 1 -> 0 – 29 years
2. Age group 2 -> 30 – 59 years
3. Age group 3 -> 60 – 79 years
4. Age group 4 -> 80+ years

Both TL and cognitive traits were Z-transformed before analyses.

### Model I

**Cognitive trait = TL + sex + age group + study specific covariates**

### Model II

**TL = GRS+ sex + age group + study specific covariates**

### Model III

**Cognitive trait = GRS + sex + age group + study specific covariates**

## Meta-analyses

Before meta-analysis, quality control was done in R, where risk score distributions were visually inspected. Inverse standard errors of the regression coefficient for each cognitive trait were plotted over the square-root of corresponding sample size and inspected for deviations from linearity that would indicate transformation- or processing errors. We assessed between-cohort heterogeneity via Cochrane’s Q-statistic and I^2^-statistics[^5-7^](#_ENREF_5). Additional sensitivity analyses were conducted for observational associations in STROOP with adjustments for smoking and alcohol in ERF and LLS1 cohorts.

## Meta-regression analyses for age

## In order to make a deeper investigation whether age was important for the TL analyses, we conducted a meta-regression analysis treating mean age as a mediator in the GRS~TL association. There seemed to be a trend with increasing effect sizes with increasing age in the cohorts as seen in **Figure S4**, but considering sample size (circles proportional to sample size) the analysis gave a non-significant result even after removing the young QIMR and the old LLS2 cohorts.

## Instrumental variable analyses

The GRS was used as instrumental variable (IV) in the Mendelian Randomization analysis, and the Wald ratio estimator was used to calculate IV-adjusted effect estimate for TL. Briefly, for each outcome, the corresponding beta from the meta-analysis of associations of GRS with the cognitive trait was divided by the beta from the meta-analysis of the association of GRS with TL: $\beta_{IV-trait}=$ $\frac{\beta_{GRS-trait}}{\beta_{GRS-TL}}$. The standard errors (SE) for the IV estimators were estimated using the delta method[^8^](#_ENREF_8):

${SE}_{IV-trait}\text{= abs}\left( \beta_{IV-trait} \right)\cdot\sqrt{\left( {{SE}_{GRS-TL}}/{\beta_{GRS-TL}} \right)^{2}+\left( {{SE}_{GRS-trait}}/{\beta_{GRS-trait}} \right)^{2}}$.

The 95% confidence intervals (CI) were calculated as: $\beta_{IV-trait}\pm1.96\cdot{SE}_{IV-trait}$. P-values were attained using the Z-statistic: $Z_{IV-trait}=\beta_{IV-trait}/{SE}_{IV-trait}$.

To further investigate if the effect size estimates were divergent, the IV estimators $\beta_{IV}$ and the observational associations $\beta_{TL-trait}$, were compared: $\beta_{Diff}=$ $\beta_{IV-trait}- \beta_{TL-trait}$. The corresponding standard error was approximated by assuming zero covariance between the estimates:

${SE}_{Diff}\text{=}\left. \sqrt{\left( {SE}_{IV-trait} \right)^{2}+\left( {SE}_{TL-trait} \right)^{2}} \right.$.

Subsequently, we used standard normal asymptotics for the difference: $Z_{Diff}=\frac{\beta_{Diff}}{{SE}_{Diff}}$ with 95% CI as: ${CI}_{Diff}=\beta_{Diff}\pm1.96 {SE}_{Diff}$. The p-value for the hypothesis $H_{0}$: $\beta_{Diff}=0$ was derived from the standard normal distribution.

## Stratifications

As a second step, we stratified the analysis based on *APOE* genotype as the ε4 allele is known to interact with cognitive performance. The definition of groups was as follows:

1. ε2/ε4 -> Exclude
2. ε4/ε4, ε4/ε3 -> Carriers
3. ε3/ε3, ε2/ε3, ε2/ε2 -> Non-carriers

Additional models were run to assess influence of *APOE* genotype and subsequently used in the IV analysis:

### Model I

In APO ε4 carriers:

**Cognitive trait = TL + sex + age group + study specific covariates**

In APO ε4 non-carriers:

**Cognitive trait = TL + sex + age group + study specific covariates**

### Model III

In APO ε4 carriers:

**Cognitive trait = GRS + sex + age group + study specific covariates**

In APO ε4 non-carriers:

**Cognitive trait = GRS + sex + age group + study specific covariates**

## Charge data analyses

Replication analyses were done using data from the CHARGE meta-analyses[^9^](#_ENREF_9)^,^ [^10^](#_ENREF_10) to investigate TL as a causal factor for general cognitive function, processing speed and executive functioning. First, 7 TL-associated SNPs used for the GRS were extracted from the CHARGE summary statistics. The individual effect of each SNP on cognitive function was plotted in the **Figure S2** with corresponding data in **Table S7**. The GRS was associated with cognitive function (β = -0.007 SD-decrease of TL per allele, 95% CI, -0.012, -0.002; p=0.006; **Figure S2; Table S7**) and was calculated as $X={\sum\beta_{i}s_{i}^{-2}}/{\sum s_{i}^{-2}}$ where β_i_ is the effect of the TL-decreasing risk alleles and s_i_ its corresponding standard error[^11^](#_ENREF_11). The IV-estimator (**Figure S2; Table S8**) was calculated as ${\beta_{GRS\_IV}=\beta}_{GRS\_CHARGE\_cognition}/\beta_{GRS\_Codd\_TL}$.

## Mendelian randomization testing using Z-score

### Z-score based Wald method for a single SNP

Here, we denote β_1_ as the effect of a SNP on the outcome with standard error being σ_1_ and β_0_ as the effect of a SNP on the exposure with standard error being σ_0_, β_iv_ and σ_iv_ as the final Mendelian randomization estimate and standard error of the exposure on the outcome. Z_1_, Z_0_, Z_iv_ are the corresponding Z-scores. Thus, we have

$$\beta_{iv}=\frac{\beta_{1}}{\beta_{0}}$$

with standard error approximated via the Delta-method as

$$\sigma_{\beta_{iv}}=\sqrt{\beta_{iv}^{2}}\sqrt{{(\frac{\sigma_{1}}{\beta_{1}})}^{2}+{(\frac{\sigma_{0}}{\beta_{0}})}^{2}}$$

Thus, the Z-score is

$$Z=\frac{\beta_{iv}}{\sigma_{\beta_{iv}}}=\frac{a}{\sqrt{{(\frac{\sigma_{1}}{\beta_{1}})}^{2}+{(\frac{\sigma_{0}}{\beta_{0}})}^{2}}}=\frac{a}{\sqrt{{(\frac{1}{Z_{1}})}^{2}+{(\frac{1}{Z_{0}})}^{2}}}$$

with a = 1 if Z_1_ and Z_0_ are in the same direction, otherwise a = -1.

### Testing the association between genetic risk score and the outcome based on Z-score

The association between GRS and the outcome is the averaged effect of each SNP on the outcome, which means the final estimate is a weighted summary of each individual SNP. Thus, we can use the same method as the authors used in the summary GWAS studies when pooling Z-scores across studies using sample sizes as the weights.

Here, we are pooling Z-scores across SNPs. The Z-score for GRS on the outcome with p SNPs and sample sizes N is thus as follows:

$$Z=\frac{\sum_{i=1}^{p} N_{i}Z_{i}}{\sum_{i=1}^{p} N_{i}}$$

### Testing the association between the exposure and the outcome based on Z-score

We can obtain the Z-score for the association between GRS on the exposure and the outcome, separately. Then, we use the equation below to obtain the final Z-score for the Wald method of the Mendelian randomization estimate test.

$$Z=\frac{a}{\sqrt{{(\frac{1}{Z_{GRS.outcome}})}^{2}+{(\frac{1}{Z_{GRS.exposure}})}^{2}}}$$

with a = 1 if Z_GRS-exposure_ and Z_GRS-outcome_ are in the same direction, otherwise a = -1.

## Acknowledgments

The Betula study was supported by grants from the Swedish Research Council (LN, RA), a Wallenberg Scholar grant from the Knut and Alice Wallenberg (KAW) Foundation and a grant from Torsten and Ragnar Söderbergs Foundation to LN. Grants to RA from the County Councils of Västerbotten, Sweden contributed to the collection and analyses of biological samples. We thank Susann Haraldsson for performing the telomere length measurements.

We thank all participants of the Leiden Longevity Study. The research leading to these results has received funding from the European Union’s Seventh Framework Programme (FP7/2007-2011) under grant agreement number 259679. This study was financially supported by the Innovation-Oriented Research Program on Genomics (SenterNovem IGE05007), the Centre for Medical Systems Biology and the Netherlands Consortium for Healthy Ageing (grant 050-060-810), all in the framework of the Netherlands Genomics Initiative, Netherlands Organization for Scientific Research (NWO), by Unilever Colworth and by BBMRI-NL, a Research Infrastructure financed by the Dutch government (NWO 184.021.007).

The work for NSHD was funded by the Medical Research Council (G0900686/1 and MC_UU_12019/1). We are very grateful to the members of this birth cohort for their continuing interest and participation in the study. We would like to acknowledge the Swallow group, UCL, who performed the DNA extractions (Rousseau, et al 2006). DOI: 10.1111/j.1469-1809.2006.00250.x. The NIHR Biomedical Research Centre at Newcastle upon Tyne Hospitals NHS Foundation Trust and Newcastle University (CMR).

We acknowledge Dr. Iiris Hovatta and Ms. Jenni Lahtinen for carrying out the FITSA telomere length analysis by qPCR. Gerontology Research Center is a joint effort between the University of Jyväskylä and the University of Tampere. The Finnish Twin study of aging (FITSA) was funded by the Ministry of Education, Academy of Finland, and the EC FP5 GenomEUtwin project. The Finnish Twin Cohort study is funded by the Academy of Finland Centre of Excellence in Complex Disease Genetics (grant numbers: 213506, 129680). We warmly thank the participating twins. The Academy of Finland (grants 213506, 129680, 263278, 265240, 292782).

E.S. is supported by Academy of Finland Post Doctoral funding (grant number 260001).

Erasmus Rucphen family study: The ERF study as a part of EUROSPAN (European Special Populations Research Network) was supported by European Commission FP6 STRP grant number 018947 (LSHG-CT-2006-01947) and also received funding from the European Community's Seventh Framework Programme (FP7/2007-2013)/grant agreement HEALTH-F4-2007-201413 by the European Commission under the programme "Quality of Life and Management of the Living Resources" of 5th Framework Programme (no. QLG2-CT-2002-01254). Najaf Amin is supported by the Netherlands Brain Foundation (project number F2013(1)-28). We are grateful to all study participants and their relatives, general practitioners and neurologists for their contributions and to P. Veraart for her help in genealogy, J. Vergeer for the supervision of the laboratory work and P. Snijders for his help in data collection.

We thank the Lothian Birth Cohort 1936 participants, and the team members who contributed to the study. Phenotype collection in the Lothian Birth Cohort 1936 was supported by Age UK (The Disconnected Mind project). REM, SEH, JMS, and IJD are members of the University of Edinburgh Centre for Cognitive Ageing and Cognitive Epidemiology (CCACE). CCACE is supported by funding from the Biotechnology and Biological Sciences Research Council (BBSRC) and the Medical Research Council (MRC) as part of the cross-council Lifelong Health and Wellbeing initiative (MR/K026992/1).

The SATSA and Gender studies were supported by NIH grants R01 AG04563, AG10175, AG028555, the MacArthur Foundation Research Network on Successful Aging, the Swedish Council for Working Life and Social Research (FAS/FORTE) (97:0147:1B, 2009-0795, 2013-2292), the Swedish Research Council (825-2007-7460, 825-2009-6141, 521-2013-8689, 2015-03255), Loo & Hans Osterman Foundation, the Foundation for Geriatric Diseases and Karolinska Institutet Delfinansiering (KID) to YZ. LG was supported by a Marie Curie intra-European Fellowship of the European Community's Seventh Framework Programme under contract number PIEF-GA-2011-300355. Telomere assessments were performed through support of a Distinguished Professor Award from the Karolinska Institutet to NLP. We thank Jenni Lahtinen for technical help in performing the telomere length measurements.

NTR warmly thanks all participants. IOF was supported by Biobanking and Biomolecular Resources Research Infrastructure (BBMRI –NL, 184.021.007).  Phenotyping and genotyping was made possible by multiple grants from  the Netherlands Organization for Scientific Research (NWO / ZonMW) 904-61-090, 985-10-002, 904-61-193,480-04-004, 400-05-717, Addiction-31160008, Middelgroot-911-09-032, Spinozapremie 56-464-14192.   We further acknowledge VU University’s Institute for Health and Care Research (EMGO+ );  Neuroscience Campus Amsterdam (NCA); the European Research Council (ERC Advanced, 230374), Rutgers University Cell and DNA Repository (NIMH U24 MH068457-06),  the Avera Institute, Sioux Falls, South Dakota (USA) and the National Institutes of Health (  NIH R01 HD042157-01A1, MH081802, Grand Opportunity grants 1RC2 MH089951 and 1RC2 MH089995). Part of the genotyping and analyses were funded by the Genetic Association Information Network (GAIN) of the Foundation for the National Institutes of Health.

NJS is supported by the British Heart Foundation and is a NIHR Senior Investigator

The Cohorts for Heart and Aging Research in Genomic Epidemiology Consortium (CHARGE) cognitive working group banner includes the authors of the following publications (in alphabetical order):

1. Davies G. et al. Genetic contributions to variation in general cognitive function: a meta-analysis of genome-wide association studies in the CHARGE consortium (N=53949). Molecular Psychiatry 20:183-192, 2015

2. Ibrahim-Verbaas CA et al. GWAS for executive function and processing speed suggests involvement of the CADM2 gene. Molecular Psychiatry 21:189-197, 2016

Hieab Adams^1,2^, David Ames^3,4^, Helene Amieva^5^, Najaf Amin^2,6^, Philippe Amouyel^7^, Nicola J Armstrong^8,9,10,11^, Amelia A Assareh^8^, John R Attia^12^, Rhoda Au^13,14^, Stefania Bandinelli^15^, James T Becker^16,17,18^, Alexa Beiser^13,14,19^, David A Bennett^20^, Claudine Berr^21,22^, Lars Bertram^23,24^, Joshua C Bis^25^, Eric Boerwinkle^26,27,28^, Ruth Boxall^29^, Jan Bressler^26^, Henry Brodaty^8,30^, Brendan M. Buckley^31^, Harry Campbell^32^, Daniel I Chasman^33^, Lori B Chibnik^34^,

Vincent Chouraki^7,13,35^, Sven Cichon^36,37,38^, Laura H Coker^39^, Janie Corley^40^, Gail Davies^40,41^, Ian J Deary^40,41^, Stephanie Debette^5,13,42^, Anton JM de Craen^2,43^, Philip L De Jager^34,44,45^, Anita L DeStefano^13,14,19^, Jingzhong Ding^46^, Carole Dufouil^5,47^, Cornelia M van Duijn^2,6^, Gudny Eiriksdottir^48^, Johan G Eriksson^49,50,51,52,53^, Thomas Espeseth^54,55^, Jessica D Faul^56^, Chloe Fawns-Ritchie^41^, Rudolf SN Fehrmann^57^, Luigi Ferrucci^58^, Annette L Fitzpatrick^59,60^, Ian Ford^61^, Myriam Fornage^26,27^, Lude Franke^57^, Melissa Garcia^62^, Generation Scotland^63^, Sudheer Giddaluru^64,65^, Rebecca F Gottesman^66^, Alan J Gow^40,41^, Hans J Grabe^67^, Michael E Griswold^68^,

Francine Grodstein^69^, Vilmunder Gudnason^48,70^, Sarah E Harris^41,71^, Tamara B Harris^62,72^, Caroline Hayward^73^, Gerardo Heiss^74^, Stefan Herms^36,37^, W David Hill^40^, Lynne J Hocking^75^,

Edith Hofer^76,77^, Per Hoffmann^36,37,38^, Albert Hofman^1,2^, Elizabeth G Holliday^12^, Yi-Chen Hsieh^78^, Jennifer Huffman^73^, Carla A Ibrahim-Verbaas^6,79^, M Arfan Ikram^1,2,79,80^, Maria K Jonsdottir^81^, J Wouter Jukema^82,83,84^, Nazanin Karbalai^85^, Sharon LR Kardia^86^, Juha Karjalainen^57^, Mirna Kirin^32^, Nicole A Kochan^8.87^, David S Knopman^88^, John B Kwok^89,90^,

Jari Lahti^49,91^, Jean-Charles Lambert^7,35^, Lenore J Launer^62,72^, Sven J van der Lee^6^, Teresa Lee^8,87^, Stephanie Le Hellard^64,65^, Guo Li^25^, Shu-Chen Li^92,93^, David C Liewald^40,41^, Ulman Lindenberger^92^, Tian Liu^23, 92^, Yongmei Liu^94^, David J Llewellyn^95^, Kurt Lohman^94^, Marisa Loitfelder^76^, Oscar L Lopez^16^, Michelle Luciano^40,41^, Astri J Lundervold^96,97,98^, Anders Lundqvist^99^, Ricardo E Marioni^29,41,100^, Karen A Mather^8^, Stela McLachlan^32^, William M Meeks^101^, Saira S Mirza^1,2^, Thomas H Mosley Jr^101^, Michael Nalls^102^, Lars-Goran Nilsson^103^,

Markus M Nöthen^37,104^, Lars Nyberg^99,105,106^, Christopher Oldmeadow^12^, Ben A Oostra^6^,

Aarno Palotie^107,108,109^, Goran Papenberg^92,110^, Alison Pattie^40,41^, Alan Penman^68^, Katja Petrovic^76^, Luke C Pilling^111^, Ozren Polasek^112^, David J Porteous^41,63,71^, Iris Postmus^2,43^,

Jacqueline F Price^32^, Bruce M Psaty^25,59,113,114^, Katri Räikkonen^91^, Paul Redmond^40^,

Ivar Reinvang^55^, Simone Reppermund^8^, Susan M Resnick^115^, Paul Ridker^33^, Lynda M Rose^33^,

Jerome I Rotter^116,117,118^, Igor Rudan^32^, Perminder S Sachdev^8,87^, Carsten O Schmidt^119^,

Helena Schmidt^76,120^, Reinhold Schmidt^76^, Peter R Schofield^121,122^, Peter W Schofield^123^,

Maaike Schuur^6,79^, Rodney J Scott^12^, Sudha Seshadri^13,14^, Joshua M Shulman^124,125^,

P Eline Slagboom^126^, Albert V Smith^48,70^, Blair H Smith^127^, Jennifer A Smith^86^, Velandai Srikanth^128,129^, John M Starr^41,130^, Vidar M Steen^64,65^, Oliver Stegle^131^, Marlene Stewart^32^,

David J Stott^132^, Qi Sun^69^, John C van Swieten^79^, Toshika Tanaka^58^, Kent D Taylor^117,133^,

Alexander Teumer^134^, Anbupalam Thalamuthu^8^, Russell Thomson^128^, Julian Trollor^8,135^,

Stella Trompet^43,82^, Stephen T Turner^136^, Andre G Uitterlinden^1,2,137^, Veronique Vitart^73^,

Jing Wang^14,19^, Galit Weinstein^13,14^, David R Weir^56^, Rudi GJ Westendorp^138^, Elisabeth Widen^108^, James F Wilson^32^, Beverly G Windham^101^, Christiane Wolf^85^, Philip A Wolf^13,14^,

Alan F Wright^73^, Margaret J Wright^139^, Kristine Yaffe^140^, Qiong Yang^14,19^, Lei Yu^20^,

Lina Zgaga^141,142^, Wei Zhao^86^

Author affiliations:

^1^Department of Epidemiology, Erasmus University Medical Center, Rotterdam, The Netherlands ^2^Netherlands Consortium for Healthy Ageing, Leiden, The Netherlands ^3^National Ageing Research Institute, Royal Melbourne Hospital, Melbourne, VIC, Australia ^4^Academic Unit for Psychiatry of Old Age, St George's Hospital, University of Melbourne, Kew, Australia ^5^Inserm U897, Epidemiology and Biostatistics, University of Bordeaux, Bordeaux, France ^6^Genetic Epidemiology Unit, Department of Epidemiology, Erasmus University Medical Center, Rotterdam, The Netherlands ^7^Inserm-UMR744, Institut Pasteur de Lille, Unité d'Epidémiologie et de Santé Publique, Lille, France ^8^Centre for Healthy Brain Ageing, School of Psychiatry, University of New South Wales, Sydney, NSW, Australia ^9^School of Mathematics and Statistics, University of Sydney, Sydney, NSW, Australia ^10^Cancer Research Program, Garvan Institute of Medical Research, Sydney, NSW, Australia ^11^School of Mathematics & Statistics and Prince of Wales Clinical School, University of New South Wales, Sydney, NSW, Australia ^12^Hunter Medical Research Institute and Faculty of Health, University of Newcastle, Newcastle, NSW, Australia ^13^Department of Neurology, Boston University School of Medicine, Boston, MA, USA ^14^Framingham Heart Study, Framingham, MA, USA ^15^Geriatric Unit, Azienda Sanitaria Firenze (ASF), Florence, Italy ^16^Department of Neurology, University of Pittsburgh, Pittsburgh, PA, USA ^17^Department of Psychiatry, University of Pittsburgh, Pittsburgh, PA, USA ^18^Department of Psychology, University of Pittsburgh, Pittsburgh, PA, USA ^19^Department of Biostatistics, Boston University School of Public Health, Boston, MA, USA ^20^Rush Alzheimer's Disease Center, Rush University Medical Center, Chicago, IL, USA ^21^Inserm, U106, Montpellier, France ^22^Université Montpellier I, Montpellier, France ^23^Max Planck Institute for Molecular Genetics, Berlin, Germany ^24^Faculty of Medicine, School of Public Health, Imperial College, London, UK ^25^Cardiovascular Health Research Unit, Department of Medicine, University of Washington, Seattle, WA, USA ^26^Human Genetics Center, School of Public Health, University of Texas Health Science Center at Houston, Houston, TX, USA ^27^Brown Foundation Institute of Molecular Medicine for the Prevention of Human Diseases, University of Texas Health Science Center at Houston, Houston, TX, USA ^28^Human Genome Sequencing Center, Baylor College of Medicine, Houston, TX, USA ^29^Medical Genetics Section, University of Edinburgh Centre for Genomic and Experimental Medicine, Institute of Genetics and Molecular Medicine, Western General Hospital, Edinburgh, UK ^30^Dementia Collaborative Research Centre, University of New South Wales, Sydney, NSW, Australia ^31^Department of Pharmacology and Therapeutics, University College Cork, Cork, Ireland ^32^Centre for Population Health Sciences, University of Edinburgh, Edinburgh, UK ^33^Division of Preventive Medicine, Brigham and Women's Hospital, Boston, MA, USA ^34^Program in Translational Neuropsychiatric Genomics, Department of Neurology, Brigham and Women's Hospital, Boston, MA, USA ^35^Inserm, U1167, Institut Pasteur de Lille, Université Lille-Nord de France, Lille, France ^36^Division of Medical Genetics, Department of Biomedicine, University of Basel, Basel, Switzerland ^37^Department of Genomics, Life and Brain Research Center, Institute of Human Genetics, University of Bonn, Bonn, Germany ^38^Institute of Neuroscience and Medicine (INM-1), Research Center Juelich, Juelich, Germany ^39^Division of Public Health Sciences and Neurology, Wake Forest School of Medicine, Winston-Salem, NC, USA ^40^Department of Psychology, University of Edinburgh, Edinburgh, UK ^41^Centre for Cognitive Ageing and Cognitive Epidemiology, University of Edinburgh, Edinburgh, UK ^42^Department of Neurology, Bordeaux University Hospital, Bordeaux, France ^43^Department of Gerontology and Geriatrics, Leiden University Medical Center, Leiden, The Netherlands ^44^Harvard Medical School, Boston, MA, USA ^45^Program in Medical and Population Genetics, Broad Institute, Cambridge, MA, USA ^46^Department of Internal Medicine, Wake Forest University School of Medicine, Winston-Salem, NC, USA ^47^Inserm U708, Neuroepidemiology, Paris, France ^48^Icelandic Heart Association, Kopavogur, Iceland ^49^Folkhälsan Research Centre, Helsinki, Finland ^50^National Institute for Health and Welfare, Helsinki, Finland ^51^Department of General Practice and Primary Health Care, University of Helsinki, Helsinki, Finland ^52^Unit of General Practice, Helsinki University Central Hospital, Helsinki, Finland ^53^Vasa Central Hospital, Vasa, Finland ^54^K.G. Jebsen Centre for Psychosis Research, Norwegian Centre For Mental Disorders Research (NORMENT), Division of Mental Health and Addiction, Oslo University Hospital and Institute of Clinical Medicine, University of Oslo, Oslo, Norway ^55^Department of Psychology, University of Oslo, Oslo, Norway ^56^Survey Research Center, Institute for Social Research, University of Michigan, Ann Arbor, MI, USA ^57^Department of Genetics, University Medical Centre Groningen, University of Groningen, Groningen, The Netherlands ^58^Translational Gerontology Branch, National Institute on Aging, Baltimore, MD, USA ^59^Department of Epidemiology, University of Washington, Seattle, WA, USA ^60^Department of Global Health, University of Washington, Seattle, WA, USA ^61^Robertson Center for Biostatistics, University of Glasgow, Glasgow, UK ^62^Laboratory of Epidemiology and Population Sciences, National Institute on Aging, Bethesda, MD, USA ^63^Generation Scotland, University of Edinburgh Centre for Genomic and Experimental Medicine, Institute of Genetics and Molecular Medicine, Western General Hospital, Edinburgh, UK ^64^K.G. Jebsen Centre for Psychosis Research and the Norwegian Centre for Mental Disorders Research (NORMENT), Department of Clinical Science, University of Bergen, Bergen, Norway ^65^Dr Einar Martens Research Group for Biological Psychiatry, Center for Medical Genetics and Molecular Medicine, Haukeland University Hospital, Bergen, Norway ^66^Department of Neurology, Johns Hopkins University School of Medicine, Baltimore, MD, USA ^67^Department of Psychiatry and Psychotherapy, University Medicine Greifswald, HELIOS-Hospital Stralsund, Stralsund, Germany ^68^Center of Biostatistics and Bioinformatics, University of Mississippi Medical Center, Jackson, MS, USA ^69^Channing Division of Network Medicine, Department of Medicine, Brigham and Women's Hospital and Harvard Medical School, Boston, MA, USA ^70^Faculty of Medicine, University of Iceland, Reykjavik, Iceland ^71^Medical Genetics Section, University of Edinburgh Centre for Genomic and Experimental Medicine, Institute of Genetics and Molecular Medicine, Western General Hospital, Edinburgh, UK ^72^Intramural Research Program National Institutes on Aging, National Institutes of Health, Bethesda, MD, USA ^73^MRC Human Genetics Unit, Institute of Genetics and Molecular Medicine, University of Edinburgh, Edinburgh, UK ^74^Department of Epidemiology, Gillings School of Global Public Health, University of North Carolina at Chapel Hill, Chapel Hill, NC, USA ^75^Division of Applied Medicine, University of Aberdeen, Aberdeen, UK ^76^Department of Neurology, Medical University of Graz, Graz, Austria ^77^Institute for Medical Informatics, Statistics and Documentation, Medical University of Graz, Graz, Austria ^78^School of Public Health, Taipei Medical University, Taipei, Taiwan ^79^Department of Neurology, Erasmus University Medical Center, Rotterdam, The Netherlands ^80^Department of Radiology, Erasmus University Medical Center, Rotterdam, The Netherlands ^81^Landspitali Hospital, Reykjavik, Iceland ^82^Department of Cardiology, Leiden University Medical Center, Leiden, The Netherlands ^83^Durrer Center for Cardiogenetic Research, Amsterdam, The Netherlands ^84^Interuniversity Cardiology Institute of the Netherlands, Utrecht, The Netherlands ^85^RG Statistical Genetics, Max Planck Institute of Psychiatry, Munich, Germany ^86^Department of Epidemiology, University of Michigan, Ann Arbor, MI, USA ^87^Neuropsychiatric Institute, Prince of Wales Hospital, Sydney, NSW, Australia ^88^Department of Neurology, Mayo Clinic, Rochester, MN, USA ^89^Neuroscience Research Australia, Randwick, NSW, Australia ^90^School of Medical Sciences, University of New South Wales, Sydney, NSW, Australia ^91^Institute of Behavioural Sciences, University of Helsinki, Helsinki, Finland ^92^Max Planck Institute for Human Development, Berlin, Germany ^93^Technische Universität Dresden, Dresden, Germany ^94^Department of Epidemiology, Wake Forest School of Medicine, Winston-Salem, NC, USA ^95^Institute of Biomedical and Clinical Sciences, University of Exeter Medical School, Exeter, UK ^96^Department of Biological and Medical Psychology, University of Bergen, Bergen, Norway ^97^Kavli Research Centre for Aging and Dementia, Haraldsplass Deaconess Hospital, Bergen, Norway ^98^K.G. Jebsen Centre for Research on Neuropsychiatric Disorders, University of Bergen, Bergen, Norway ^99^Umeå Center for Functional Brain Imaging (UFBI), Umeå University, Umeå, Sweden ^100^Queensland Brain Institute, The University of Queensland, Brisbane, QLD, Australia ^101^Department of Medicine, Division of Geriatrics, University of Mississippi Medical Center, Jackson, MS, USA ^102^Laboratory of Neurogenetics, National Institute on Aging, Bethesda, MD, USA ^103^ARC, Karolinska Institutet, Stockholm and UFBI, Umeå University, Umeå, Sweden ^104^German Center for Neurodegenerative Diseases (DZNE), Bonn, Germany ^105^Department of Radiation Sciences, Umeå University, Umeå, Sweden ^106^Department of Integrative Medical Biology, Umeå University, Umeå, Sweden ^107^Wellcome Trust Sanger Institute, Wellcome Trust Genome Campus, Cambridge, UK ^108^Institute for Molecular Medicine Finland (FIMM), University of Helsinki, Helsinki, Finland ^109^Department of Medical Genetics, University of Helsinki and University Central Hospital, Helsinki, Finland ^110^Karolinska Institutet, Aging Research Center, Stockholm University, Stockholm, Sweden ^111^Epidemiology and Public Health Group, University of Exeter Medical School, Exeter, UK ^112^Faculty of Medicine, Department of Public Health, University of Split, Split, Croatia ^113^Deparment of Health Services, University of Washington, Seattle, WA, USA ^114^Group Health Research Unit, Group Health Cooperative, Seattle, WA, USA ^115^Laboratory of Behavioral Neuroscience, National Institute on Aging, NIH, Baltimore, MD, USA ^116^Medical Genetics Institute, Cedars-Sinai Medical Center, Los Angeles, CA, USA ^117^Institute for Translational Genomics and Population Sciences, Los Angeles BioMedical Research Institute at Harbor-UCLA Medical Center, Torrance, CA, USA ^118^Division of Genetic Outcomes, Department of Pediatrics, Harbor-UCLA Medical Center, Torrance, CA, USA ^119^Institute for Community Medicine, University Medicine Greifswald, Greifswald, Germany ^120^Centre for Molecular Medicine, Institute of Molecular Biology and Biochemistry, Medical University of Graz, Graz, Austria ^121^Neuroscience Research Australia, Sydney, NSW, Australia ^122^Faculty of Medicine, University of New South Wales, Sydney, NSW, Australia ^123^School of Medicine and Public Health, University of Newcastle, Newcastle, NSW, Australia ^124^Department of Neurology, Baylor College of Medicine, Houston, TX, USA ^125^Department of Molecular and Human Genetics, The Jan and Dan Duncan Neurological Research Institute, Texas Children's Hospital, Houston, TX, USA ^126^Department of Molecular Epidemiology, Leiden University Medical Center, Leiden, The Netherlands ^127^Medical Research Institute, University of Dundee, Dundee, UK ^128^Menzies Research Institute, Hobart, Tasmania ^129^Stroke and Ageing Research, Medicine, Southern Clinical School, Monash University, Melbourne, VIC, Australia ^130^Alzheimer Scotland Dementia Research Centre, University of Edinburgh, Edinburgh, UK ^131^Max Planck Institute for Developmental Biology, Max Planck Institute for Intelligent Systems, Tübingen, Germany ^132^Institute of Cardiovascular and Medical Sciences, University of Glasgow, Glasgow, UK ^133^Department of Pediatrics, Harbor-UCLA Medical Center, Los Angeles, CA, USA ^134^Interfaculty Institute for Genetics and Functional Genomics, University Medicine Greifswald, Greifswald, Germany ^135^Department of Developmental Disability Neuropsychiatry, School of Psychiatry, University of New South Wales, Sydney, NSW, Australia ^136^Division of Nephrology and Hypertension, Department of Internal Medicine, Mayo Clinic, Rochester, MN, USA ^137^Department of Internal Medicine, Erasmus University Medical Center, Rotterdam, The Netherlands ^138^Leiden Academy of Vitality and Ageing, Leiden, The Netherlands ^139^Neuroimaging Genetics Group, QIMR Berghofer Medical Research Institute, Brisbane, QLD, Australia ^140^Departments of Psychiatry, Neurology and Epidemiology, University of California, San Francisco and San Francisco VA Medical Center, San Francisco, CA, USA ^141^Department of Public Health and Primary Care, Trinity College Dublin, Dublin, Ireland ^142^Andrija Stampar School of Public Health, Medical School, University of Zagreb, Zagreb, Croatia

Correspondence to the NeuroCHARGE cognitive working group:

Jan Bressler

Email: [jan.bressler@uth.tmc.edu](mailto:jan.bressler@uth.tmc.edu)

CHARGE Consortium: Infrastructure for the CHARGE Consortium is supported in part by the National Heart, Lung and Blood Institute grant HL105756 and for the neuroCHARGE phenotype working group through the National Institute on Aging grant AG033193.

Age, Gene/Environment Susceptibility-Reykjavik Study (AGES-Reykjavik): This study has been funded by NIH contracts N01-AG-12100 and 271201200022C, the NIA Intramural Research Program, Hjartavernd (the Icelandic Heart Association), and the Althingi (the Icelandic Parliament) with contributions from NEI, NIDCD and NHLBI. The study was approved by the Icelandic National Bioethics Committee (VSN: 00-063). The researchers are indebted to the participants for their willingness to participate in the study.

Aspirin for Asymptomatic Atherosclerosis Study (AAA): We thank the cohort participants and team members who contributed to this study. Phenotype collection and DNA extraction were supported by the Wellcome Trust, the British Heart Foundation and the Chief Scientist Office of the Scottish Executive. The AAA Trial was performed and the database is maintained by members of the University of Edinburgh Molecular Epidemiology Research Group in the Centre for Population Health Sciences. We also thank staff at the Wellcome Trust Clinical Research Facility in Edinburgh where some of the research clinics and genotyping were undertaken.

Atherosclerosis Risk in Communities (ARIC) Study: The Atherosclerosis Risk in Communities Study is carried out as a collaborative study supported by National Heart, Lung and Blood Institute Contracts (HHSN268201100005C, HHSN268201100006C, HHSN268201100007C, HHSN268201100008C, HHSN268201100009C, HHSN268201100010C, HHSN268201100011C and HHSN268201100012C), R01HL70825, R01HL087641, R01HL59367 and R01HL086694; National Human Genome Research Institute Contract U01HG004402; and National Institutes of Health Contract HHSN268200625226C. The authors thank the staff and participants of the ARIC study for their important contributions. Infrastructure was partly supported by Grant Number UL1RR025005, a component of the National Institutes of Health and NIH Roadmap for Medical Research.

Austrian Stroke Prevention Study (ASPS): The ASPS is funded by the Austrian Science Fond (FWF) grant number P20545-P05 and P13180. The Medical University of Graz supports the databank of ASPS.

Baltimore Longitudinal Study of Aging (BLSA): The Baltimore Longitudinal Study of Aging is supported by the Intramural Research Program of the NIH, National Institute on Aging.

Berlin Aging Study (BASE-II): BASE-II has been financed by the Max Planck Society and the Federal Ministry of Education and Research. For a summary of the design of the study, see Bertram, L., Böckenhoff, A., Demuth, I., Düzel, S., Eckardt, R., Li, S.-C., Lindenberger, U., Pawelec, G., Siedler, T., Wagner, G. G., & Steinhagen-44 Thiessen, E. (2013). Cohort profile: The Berlin Aging Study II (BASE-II). Advance online publication. International Journal of Epidemiology.

Betula Study (BETULA): The Betula Study was supported by the Swedish Research Council to Lars-Göran Nilsson and Lars Nyberg (2001-6654, 2002-3794 and 2003-3883) and by a Wallenberg Scholar grant from the Knut and Alice Wallenberg Foundation to Lars Nyberg. Sudheer Giddaluru was supported by a grant from Helse Vest RHF to Stephanie Le Hellard (Grant 911554). We also thank the Centre for Advanced Study (CAS) at the Norwegian Academy of Science and Letters in Oslo for hosting collaborative projects and workshops between Norway, Sweden and Scotland in 2011-2012.

Cardiovascular Health Study (CHS): This research was supported by NHLBI contracts HHSN268201200036C, HHSN268200800007C, N01HC55222, N01HC85079, N01HC85080, N01HC85081, N01HC85082, N01HC85083, N01HC85086; and NHLBI grants U01HL080295, R01HL087652, R01HL105756, R01HL103612, R01HL120393, and R01HL130114 with additional contribution from the National Institute of Neurological Disorders and Stroke (NINDS). Additional support was provided through R01AG023629, R01AG15928, R01AG20098, R01AG05133, and R01AG027058 from the National Institute on Aging (NIA). A full list of principal CHS investigators and institutions can be found at http://CHS-NHLBI.org. The provision of genotyping data was supported in part by the National Center for Advancing Translational Sciences, CTSI grant UL1TR000124, and the National Institute of Diabetes and Digestive and Kidney Disease Diabetes Research Center (DRC) grant DK063491 to the Southern California Diabetes Endocrinology Research Center. The content is solely the responsibility of the authors and does not necessarily represent the official views of the National Institutes of Health.

CROATIA-Korcula: The CROATIA-Korcula study was funded by grants from the Medical Research Council (UK), European Commission Framework 6 project EUROSPAN (Contract No. LSHG-CT-2006-018947) and Republic of Croatia Ministry of Science, Education and Sports research grants to IR (108-1080315-0302). We would like to acknowledge the invaluable contributions of the recruitment team in Korcula, the administrative teams in Croatia and Edinburgh and the people of Korcula. The SNP genotyping for the CROATIA-Korcula cohort was performed in Helmholtz Zentrum München, Neuherberg, Germany.

CROATIA-Split: The CROATIA-Split study is funded by grants from the Medical Research Council (UK), European Commission Framework 6 project EUROSPAN (Contract No. LSHG-CT-2006-018947) and Republic of Croatia Ministry of Science, Education and Sports research grants to IR (108-1080315-0302). We would like to acknowledge the invaluable contributions of the recruitment team in Split, the administrative teams in Croatia and Edinburgh and the people of Split. The SNP genotyping for the CROATIA-Split cohort was performed by AROS Applied Biotechnology, Aarhus, Denmark.

CROATIA-Vis: The CROATIA-Vis study was funded by grants from the Medical Research Council (UK) and Republic of Croatia Ministry of Science, Education and Sports research grants to IR (108-1080315-0302). We would like to acknowledge the staff of several institutions in Croatia that supported the field work, including but not limited to The University of Split and Zagreb Medical Schools, the Institute for Anthropological Research in Zagreb and Croatian Institute for Public Health. The SNP genotyping for the CROATIA-Vis cohort was performed in the core genotyping laboratory of the Wellcome Trust Clinical Research Facility at the Western General Hospital, Edinburgh, Scotland.

Dynamics of Health, Aging and Body Composition (Health ABC): This research was supported by NIA Contracts N01AG62101, N01AG62103 and N01AG62106. The genome-wide association study was funded by NIA Grant 1R01AG032098-01A1 to Wake Forest University Health Sciences and genotyping services were provided by the Center for Inherited Disease Research (CIDR). CIDR is fully funded through a federal contract from the National Institutes of Health to The Johns Hopkins University, Contract Number HHSN268200782096C. This research was supported in part by the Intramural Research Program of the NIH, National Institute on Aging.

Erasmus Rucphen Family Study (ERF): The ERF study as a part of EUROSPAN (European Special Populations Research Network) was supported by European Commission FP6 STRP grant number 018947 (LSHG-CT-2006-01947) and also received funding from the European Community’s Seventh Framework Programme (FP7/2007-2013)/grant agreement HEALTH-F4-2007-201413 by the European Commission under the programme “Quality of Life and Management of the Living Resources” of 5th Framework Programme (no. QLG2-CT-2002-01254). This study was financially supported by the Netherlands Organization for Scientific Research (NWO), the Internationale Stichting Alzheimer Onderzoek (ISAO), the Hersenstichting Nederland (HSN) and the Centre for Medical Systems Biology (CMSB) in the framework of the Netherlands Genomics Initiative (NGI) and by the Russian Foundation for Basic Research (RFBR). We thank the participants from the Genetic Research in Isolated Populations, Erasmus Rucphen Family, who made this work possible. Also, we thank Petra Veraart for collecting all genealogical data.

Framingham Heart Study (FHS): This work was supported by the dedication of the Framingham Study participants, the National Heart, Lung and Blood Institute's Framingham Heart Study (Contract No. HHSN268201500001I), and by grants from the National Institute of Health (R01s AG008122, AG054076, AG049607, AG033193, AG010129, AG033040, NS017950, and U01AG49505). The content is solely the responsibility of the authors and does not necessarily represent the official views of the National Institutes of Health.

Generation Scotland: Scottish Family Health Study (GS): Generation Scotland has received core funding from Chief Scientist Office of the Scottish Government Health Directorates ZD/16/6and the Scottish Funding Council HR03006. We are grateful to all the families who took part, the general practitioners and the Scottish School of Primary Care for their help in recruiting them, and the whole Generation Scotland team, which includes interviewers, computer and laboratory technicians, clerical workers, research scientists, volunteers, managers, receptionists, healthcare assistants and nurses. Genotyping of the GS:SFHS samples was carried out by the Genetics Core Laboratory at the Wellcome Trust Clinical Research Facility, Edinburgh, Scotland and was funded by the UK’s Medical Research Council. REM and DJP undertook the work within the University of Edinburgh Centre for Cognitive Ageing and Cognitive Epidemiology, part of the cross council Lifelong Health and Wellbeing Initiative (MR/K026992/1). Funding from BBSRC and Medical Research Council (MSRC) is gratefully acknowledged.

Genetic Epidemiology Network of Arteriopathy (GENOA): Support for the Genetic Epidemiology Network of Arteriopathy (GENOA) was provided by the National Heart, Lung and Blood Institute (HL054464, HL054457, HL054481, HL071917 and HL87660) and the National Institute of Neurological Disorders and Stroke (NS041558) of the National Institutes of Health. Genotyping was performed at the Mayo Clinic (Stephen T. Turner, Mariza de Andrade, Julie Cunningham) and was made possible by the University of Texas Health Sciences Center (Eric Boerwinkle, Megan L Grove-Gaona). We would also like to thank the families that participated in the GENOA study.

Health and Retirement Study (HRS): HRS is supported by the National Institute on Aging (NIA U01AG009740). The genotyping was funded separately by the National Institute on Aging (RC2 AG036495, RC4 AG039029). Our genotyping was conducted by the NIH Center for Inherited Disease Research (CIDR) at Johns Hopkins University. Genotyping quality control and final preparation of the data were performed by the Genetics Coordinating Center at the University of Washington.

Helsinki Birth Cohort Study (HBCS): We thank all study participants as well as everybody involved in the Helsinki Birth Cohort Study. Helsinki Birth Cohort Study has been supported by grants from the Academy of Finland, the Finnish Diabetes Research Society, Folkhälsan Research Foundation, Novo Nordisk Foundation, Finska Läkaresällskapet, Signe and Ane Gyllenberg Foundation, University of Helsinki, Ministry of Education, Ahokas Foundation, Emil Aaltonen Foundation, Juho Vainio Foundation and Wellcome Trust (Grant Number WT089062).

Hunter Community Study (HCS): The authors would like to thank the men and women participating in the HCS as well as all the staff, investigators and collaborators who have supported or been involved in the project to date. The cohort was made possible with support from the University of Newcastle's Strategic Initiative Fund, the Vincent Fairfax Family Foundation and the Hunter Medical Research Institute.

InCHIANTI: The Invechhiare in Chianti (InCHIANTI) Study was supported as a targeted project (ICS 110.1RS97.71) by the Italian Ministry of Health, by the US National Institute on Aging (Contracts N01[AG]916413, N01[AG] 821336, 263 MD 9164 13, and 263 MD 821336), and, in part, by the Intramural Research Program, National Institute on Aging, National Institutes of Health.

Lothian Birth Cohort 1921 (LBC1921) and Lothian Birth Cohort 1936 (LBC1936): We thank the cohort participants and team members who contributed to these studies. Phenotype collection in the Lothian Birth Cohort 1921 was supported by the UK Biotechnology and Biological Sciences Research Council (BBSRC), The Royal Society and The Chief Scientist Office of the Scottish Government. Phenotype collection in the Lothian Birth Cohort 1936 was supported by Research Into Ageing (continues as part of Age UK The Disconnected Mind project). Genotyping of the cohorts was funded by the BBSRC. The work was undertaken by The University of Edinburgh Centre for Cognitive Ageing and Cognitive Epidemiology, part of the cross council Lifelong Health and Wellbeing Initiative (MR/K026992/1). Funding from the BBSRC and Medical Research Council (MRC) is gratefully acknowledged.

Norwegian Cognitive NeuroGenetics Cohort (NCNG): The NCNG study has been funded through the Research Council of Norway (including the FUGE program), the National Institutes of Health, the University of Oslo, the University of Bergen, the Bergen Research Foundation (BFS), Helse Vest, and the Western Norway Regional Health Authority, the KG Jebsen Centre for Psychosis Research, and Dr. Einar Martens Fund. We also thank the Centre for Advanced Study (CAS) at the Norwegian Academy of Science and Letters in Oslo for hosting collaborative projects and workshops between Norway, Sweden and Scotland in 2011-2012.

Nurses’ Health Study (NHS): This study was supported by research Grants CA87969, CA49449, HL34594, U01HG004399, DK058845, CA65725, CA67262, CA50385, 5UO1CA098233, EY09611, EY015473, HG004728, HL35464, CA55075, CA134958 and DK070756 from the National Institutes of Health. The genotyping was partly supported by an unrestricted grant from Merck Research Laboratories. Dr. Sun is supported by career development award K99HL098459 from the National Heart, Lung and Blood Institute.

Older Australian Twins Study (OATS): We thank the OATS participants and gratefully acknowledge the support and assistance of the OATS Research Team. This work was facilitated by access to the Australian Twin Registry, a national research resource supported by the NHMRC Enabling Grant 310667 and administered by the University of Melbourne. DNA was extracted by Genetic Repositories Australia, an Enabling Facility, supported by the NHMRC Grant 401184. OATS genotyping was partly funded by a CSIRO Flagship Collaboration Fund Grant. Genome-wide genotyping was performed by the Diamantina Institute, University of Queensland. OATS is supported by the National Health and Medical Research Council of Australia (NHMRC) Project Grant 1045325 and the NHMRC/ARC Strategic Award 401162.

Orkney Complex Disease Study (ORCADES): ORCADES was supported by the Chief Scientist Office of the Scottish Government, the Royal Society, the MRC Human Genetics Unit, Arthritis Research UK and the European Union framework program 6 EUROSPAN project (Contract No. LSHG-CT-2006-018947). DNA extractions were performed at the Wellcome Trust Clinical Research Facility in Edinburgh. We would like to acknowledge the invaluable contributions of Lorraine Anderson and the research nurses in Orkney, the administrative team in Edinburgh and the people of Orkney.

PROspective Study of Pravastatin in the Elderly at Risk (PROSPER): The PROSPER study was supported by an investigator initiated grant obtained from Bristol-Myers Squibb. Prof. Dr. JW Jukema is an Established Clinical Investigator of the Netherlands Heart Foundation (Grant 2001 D 032). Support for genotyping was provided by the seventh framework program of the European commission (Grant 223004) and by the Netherlands Genomics Initiative (Netherlands Consortium for Healthy Aging Grant 050-060-810).

Religious Orders Study (ROS) and Rush Memory and Aging Project (MAP): The ROS and MAP data in the analysis is supported by National Institute on Aging grants P30AG10161, R01AG17917, R01AG15819, R01AG30146, the Illinois Department of Public Health, and the Translational Genomics Research Institute.

Rotterdam Study (RS): The Rotterdam Study is funded by Erasmus Medical Center and Erasmus University, Rotterdam, Netherlands Organization for the Health Research and Development (ZonMw), the Research Institute for Diseases in the Elderly (RIDE), the Ministry of Education, Culture and Science, the Ministry for Health, Welfare and Sports, the European Commission (DG XII), and the Municipality of Rotterdam. The generation and management of GWAS genotype data for the Rotterdam Study (RS I, RS II, RS III) were executed by the Human Genotyping Facility of the Genetic Laboratory of the Department of Internal Medicine, Erasmus MC, Rotterdam, The Netherlands. The GWAS datasets are supported by the Netherlands Organisation of Scientific Research NWO Investments (nr. 175.010.2005.011, 911-03-012), the Genetic Laboratory of the Department of Internal Medicine, Erasmus MC, the Research Institute for Diseases in the Elderly (014-93-015; RIDE2), the Netherlands Genomics Initiative (NGI)/Netherlands Organisation for Scientific Research (NWO) Netherlands Consortium for Healthy Aging (NCHA), project nr. 050-060-810. We thank Pascal Arp, Mila Jhamai, Marijn Verkerk, Lizbeth Herrera and Marjolein Peters, and Carolina Medina-Gomez, for their help in creating the GWAS database, and Karol Estrada, Yurii Aulchenko, and Carolina Medina-Gomez, for the creation and analysis of imputed data. The authors are grateful to the study participants, the staff from the Rotterdam Study and the participating general practitioners and pharmacists.

Study of Health in Pomerania (SHIP-TREND): SHIP is part of the Community Medicine Research net of the University Medicine of Greifswald, Germany, which is funded by the Federal Ministry of Education and Research (grants no. 01ZZ9603, 01ZZ0103, and 01ZZ0403), the Ministry of Cultural Affairs as well as the Social Ministry of the Federal State of Mecklenburg-West Pomerania. The SHIP authors are grateful to Holger Prokisch and Thomas Meitinger (Helmholtz Zentrum München) for the genotyping of the SHIP-TREND cohort.

Sydney Memory and Ageing Study (MAS): We would like to acknowledge and thank the Sydney MAS participants and the Research Team for their contributions and assistance. DNA was extracted by Genetic Repositories Australia, an Enabling Facility supported by NHMRC Grant 401184. Genotyping was performed by the Ramaciotti Centre, University of New South Wales. Sydney MAS is supported by the Australian National Health& Medical Research Council Program Grants 350833, 568969, and 109308. Karen Mather is supported by the Capacity Building Grant 568940. NAK is also supported by a NHMRC Early Career Fellowship.

Tasmanian Study of Gait and Cognition (TASCOG): TASCOG is supported by the National Health and Medical Research Council of Australia (NHMRC) Project Grants 403000, 491109; National Heart Foundation/NHMRC Career Development Fellowship 606544 (VS); NHMRC Career Development Fellowship (RS).

Three-City Study (3CS): We thank the staff and participants of the 3C Study for their important contributions. The 3C Study is conducted under a partnership agreement between INSERM, Victor Segalen–Bordeaux II University and Sanofi-Aventis. The Fondation pour la Recherche Médicale funded the preparation and initiation of the study. The 3C Study is also supported by the Caisse Nationale Maladie des Travailleurs Salariés, Direction Générale de la Santé, Mutuelle Générale de l’Education Nationale (MGEN), Institut de la Longévité, Conseils Régionaux de Aquitaine et Bourgogne, Fondation de France and the French Ministry of Research–INSERM Programme Cohortes et Collections de Données Biologiques. This work was supported by the National Foundation for Alzheimer’s Disease and Related Disorders, the Institut Pasteur de Lille, the Centre National de Génotypage and the LABEX (Laboratory of Excellence program investment for the future) DISTALZ - Development of Innovative Strategies for a Transdisciplinary approach to ALZheimer’s disease.

Women’s Genome Health Study (WGHS): The WGHS is supported by HL043851 and HL080467 from the National Heart, Lung and Blood Institute and CA047988 from the National Cancer Institute, the Donald W Reynolds Foundation and the Fondation Leducq, with collaborative scientific support and funding for genotyping provided by Amgen.

## References

1. Codd V, Nelson CP, Albrecht E, Mangino M, Deelen J, Buxton JL *et al.* Identification of seven loci affecting mean telomere length and their association with disease. *Nat Genet* 2013; **45**(4)**:** 422-427.

2. Lawlor DA. Commentary: Two-sample Mendelian randomization: opportunities and challenges. *Int J Epidemiol* 2016; **45**(3)**:** 908-915.

3. Johnson T. Efficient Calculation for Multi-SNP Genetic Risk Scores. *ASHG poster, San Francisco* 2012.

4. gtx: Genetics ToolboX (R-package). https://cran.r-project.org/web/packages/gtx/, 2012, Accessed Date Accessed 2012 Accessed.

5. Higgins JP, Thompson SG. Quantifying heterogeneity in a meta-analysis. *Stat Med* 2002; **21**(11)**:** 1539-1558.

6. Higgins JP, Thompson SG, Deeks JJ, Altman DG. Measuring inconsistency in meta-analyses. *Bmj* 2003; **327**(7414)**:** 557-560.

7. Ioannidis JP, Patsopoulos NA, Evangelou E. Uncertainty in heterogeneity estimates in meta-analyses. *Bmj* 2007; **335**(7626)**:** 914-916.

8. Oehlert GW. A note on the delta method. *American Statistician* 1992; **46:** 27-29.

9. Davies G, Armstrong N, Bis JC, Bressler J, Chouraki V, Giddaluru S *et al.* Genetic contributions to variation in general cognitive function: a meta-analysis of genome-wide association studies in the CHARGE consortium (N=53949). *Mol Psychiatry* 2015; **20**(2)**:** 183-192.

10. Ibrahim-Verbaas CA, Bressler J, Debette S, Schuur M, Smith AV, Bis JC *et al.* GWAS for executive function and processing speed suggests involvement of the CADM2 gene. *Mol Psychiatry* 2016; **21**(2)**:** 189-197.

11. Dastani Z, Johnson T, Kronenberg F, Nelson CP, Assimes TL, Marz W *et al.* The shared allelic architecture of adiponectin levels and coronary artery disease. *Atherosclerosis* 2013; **229**(1)**:** 145-148.

## Figure Legends

Supplementary Figure 1. Meta-analysis of genetic risk score association with telomere length across all European ancestry cohorts (N=17 052). The seven SNPs included in the risk score were all identified in the largest telomere length genome-wide association study to date (Codd et al, 2013).

Supplementary Figure 2. Associations of the seven SNPs and the combined risk score with telomere length from Codd et al, 2013, (left panel) and with general cognitive performance in CHARGE data with N=53 949 from Davies et al, 2015, (middle panel). Right panel includes the full instrumental variable analysis providing a causal estimate for telomere length on general cognitive performance.

Supplementary Figure 3. Scatter plots showing the per-allele association from CHARGE summary data with cognitive performance traits (general cognitive performance, animal naming, LF, trails A, and trails B) plotted against the per-allele association with telomere length (with vertical lines showing 95% confidence interval for each SNP). Because the effect sizes of SNPs on DSST and Stroop are unavailable, we could not perform such pleiotropy test using the same method as for general cognitive performance.

Supplementary Figure 4. Scatter plot showing the regression coefficient for GRS~TL over mean age in cohorts. Area of circle is proportional to number of subjects in study, line is a loess smoothing curve.

Supplementary Figure 1

Supplementary Figure 2


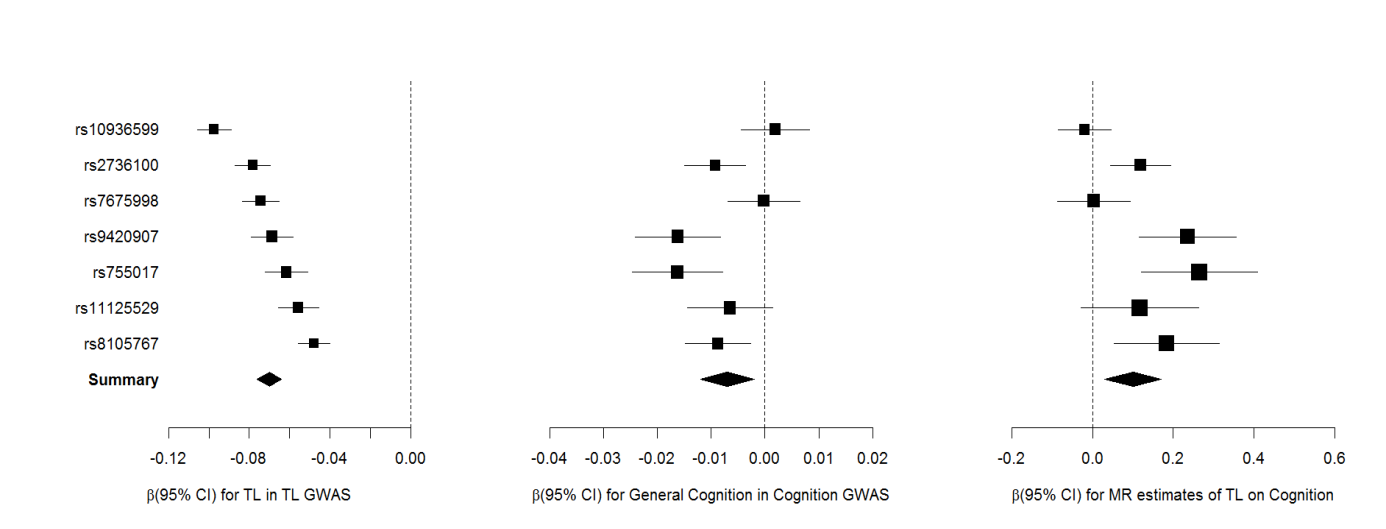


Supplementary Figure 3


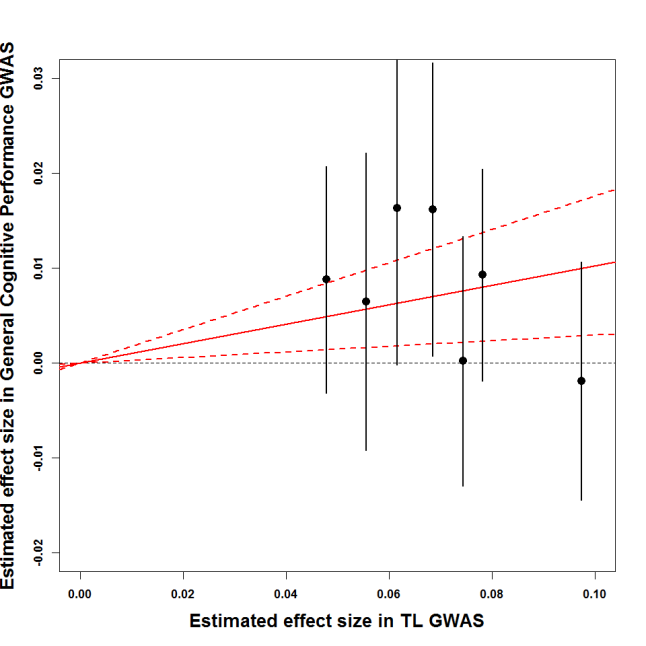

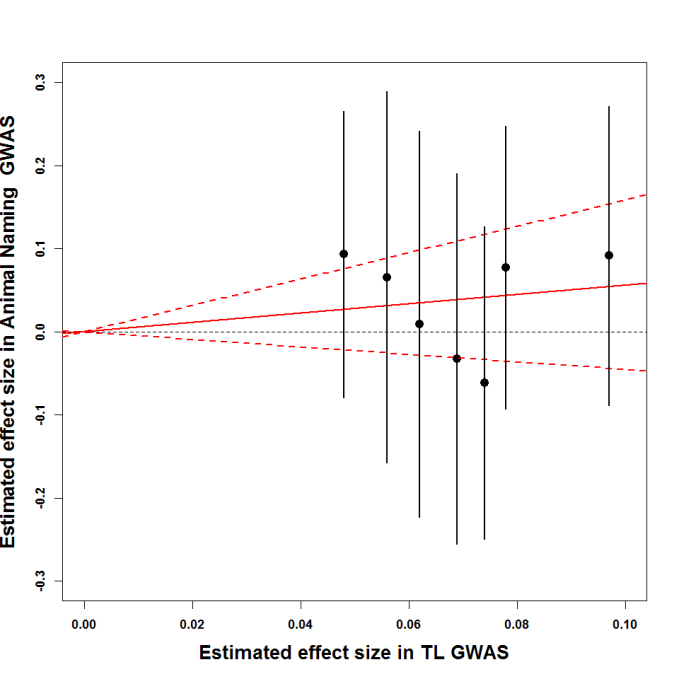

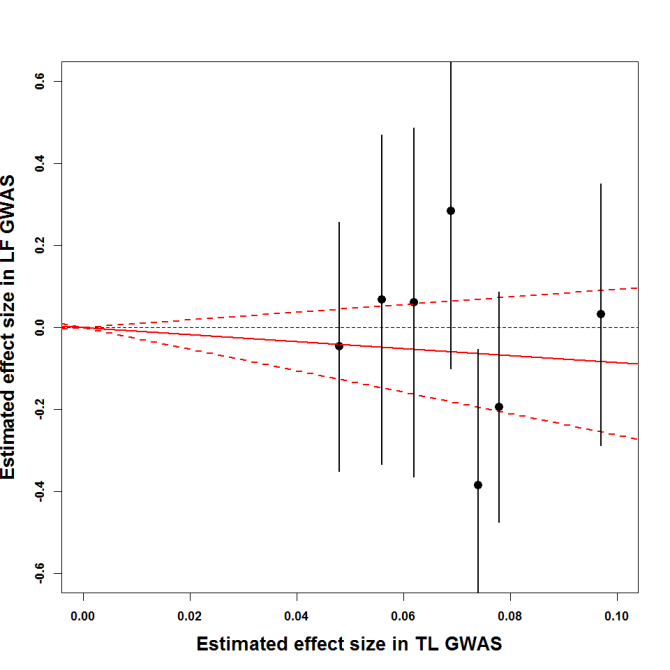

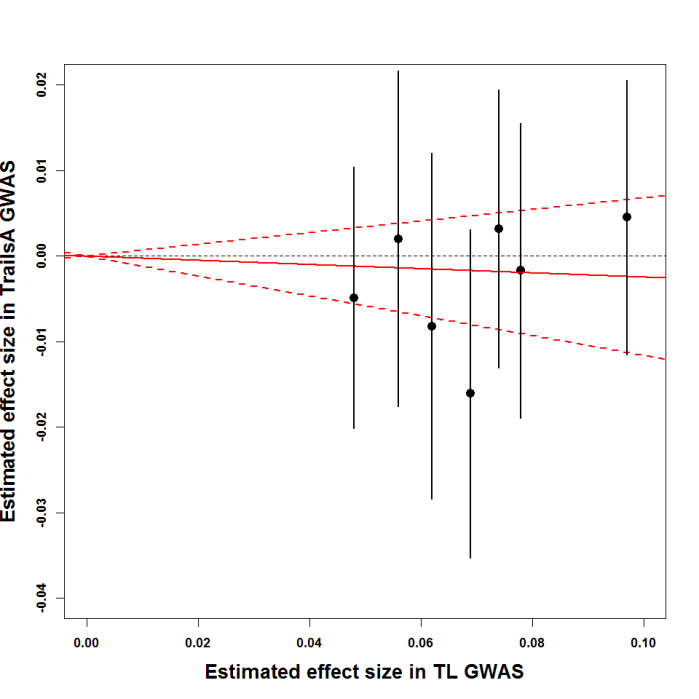

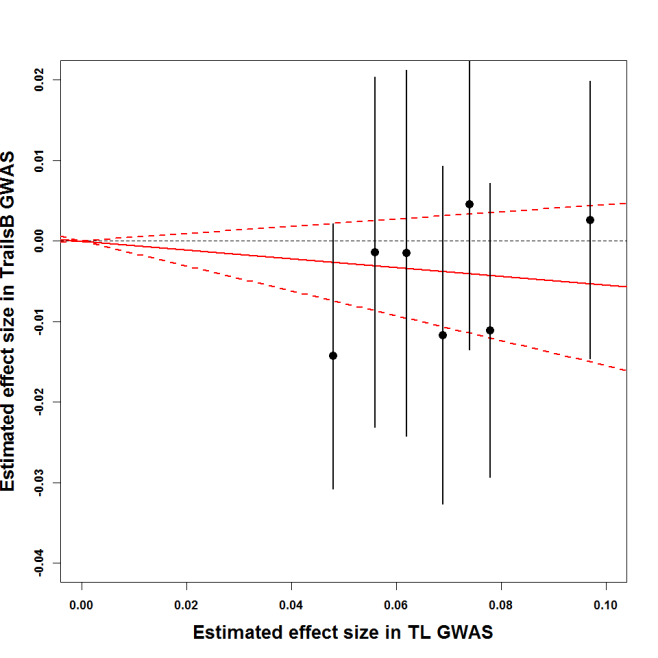


Supplementary Figure 4.


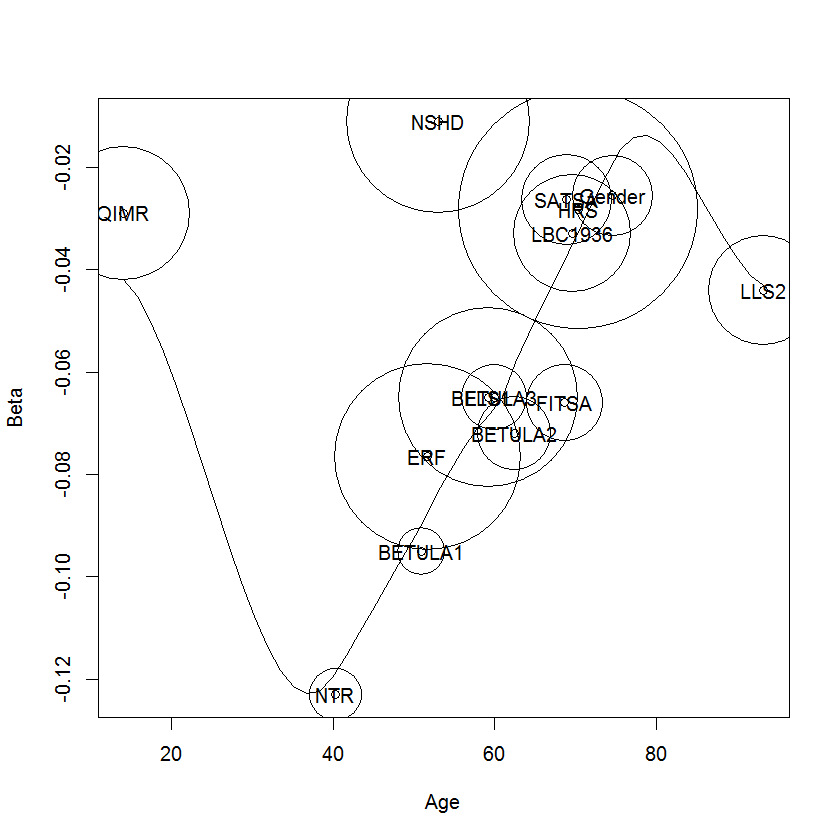

Supplement: Supplementary Information [file tp201773x1.docx]
